# Ethnopharmacology, Antimicrobial Potency, and Phytochemistry of African *Combretum* and *Pteleopsis* Species (Combretaceae): A Review

**DOI:** 10.3390/antibiotics12020264

**Published:** 2023-01-28

**Authors:** Heidi Silén, Enass Y. A. Salih, Eunice Ego Mgbeahuruike, Pia Fyhrqvist

**Affiliations:** Division of Pharmaceutical Biosciences, Faculty of Pharmacy, University of Helsinki, 00014 Helsinki, Finland

**Keywords:** *Combretum*, *Pteleopsis*, antibacterial, antifungal, traditional medicine, antibiotic adjuvants

## Abstract

Bacterial and fungal resistance to antibiotics is of growing global concern. Plants such as the African *Combretum* and *Pteleopsis* species, which are used in traditional medicine for the treatment of infections, could be good sources for antimicrobial extracts, drug scaffolds, and/or antibiotic adjuvants. In African countries, plant species are often used in combinations as traditional remedies. It is suggested that the plant species enhance the effects of each other in these combination treatments. Thus, the multi-species-containing herbal medications could have a good antimicrobial potency. In addition, plant extracts and compounds are known to potentiate the effects of antibiotics. The objective of this review is to compile the information on the botany, ethnopharmacology, ethnobotany, and appearance in herbal markets of African species of the genera *Combretum* and *Pteleopsis*. With this ethnobotanical information as a background, this review summarizes the information on the phytochemistry and antimicrobial potency of the extracts and their active compounds, as well as their combination effects with conventional antibiotics. The databases used for the literature search were Scopus, Elsevier, EBSCOhost, PubMed, Google Scholar, and SciFinder. In summary, a number of *Combretum* and *Pteleopsis* species were reported to display significant in vitro antibacterial and antifungal efficacy. Tannins, terpenes, flavonoids, stilbenes, and alkaloids—some of them with good antimicrobial potential—are known from species of the genera *Combretum* and *Pteleopsis*. Among the most potent antimicrobial compounds are arjunglucoside I (MIC 1.9 µg/mL) and imberbic acid (MIC 1.56 µg/mL), found in both genera and in some *Combretum* species, respectively. The in vitro antimicrobial properties of the extracts and compounds of many *Combretum* and *Pteleopsis* species support their traditional medicinal uses.

## 1. Introduction

Bacterial and fungal resistance to antibiotics is of growing global concern [1]. Drug-resistant tuberculosis (TB), the number one infectious-disease killer globally, causes 1.8 million deaths per year, and there are only a limited number of successful second-line treatments against multi-drug-resistant- (MDR-TB) and extensively-drug-resistant- (XDR-TB) tuberculosis [2]. Other important, antibiotic-resistant bacteria include methicillin-resistant *Staphylococcus aureus* (MRSA), vancomycin-resistant *Enterococcus* (VRE), and carbapenem-resistant Enterobacteriaceae (CRE) [1]. In addition, the incidence of antibiotic resistance is increasing among bacteria, causing serious diarrhea and sepsis, such as with *Clostridioides difficile*, *Escherichia coli*, and *Klebsiella pneumoniae* [3,4]. *Candida glabrata* has demonstrated an increased significance among the human-pathogenic isolates of the *Candida* species, and many clinical isolates of *C. glabrata* are reported to be more resistant to amphotericin-B than *C. albicans* [5]. In their report on new, approved antibiotics in the drug pipeline, the WHO [3] noted that most of these antibiotics are derivatives of known classes, and therefore a fast development of emerging resistance against these agents is foreseen. There is a need for new antibiotic treatments, including combination therapies, to combat resistant bacteria and fungi. Higher plants used in traditional medicine for the treatment of bacterial and fungal infections could be potential sources of antibiotic potentiators (adjuvants) and new antibiotic scaffolds [6,7].

This review focuses on the African species of the genera *Combretum* and *Pteleopsis,* which could play an important role in the development of new antibiotic scaffolds and/or antibiotic adjuvants. In Africa, plants from various regions have been used as medicine since ancient times, including for the treatment of infections and their symptoms [8]. Depending on the country and the region (countryside or city), approximately 60–80% of the people in Africa utilize plants as their primary form of medical treatment [9]. Although a number of extracts and compounds from African medicinal plants have been reported to possess promising antimicrobial potential, either alone or as antibiotic adjuvants, only a small proportion of the drugs derived from these plants have been marketed globally, and none of these drugs are antimicrobials [10]. Instead, extracts and compounds are utilized commercially as antioxidants and skin creams, among other uses [11].

**Figure 1:**
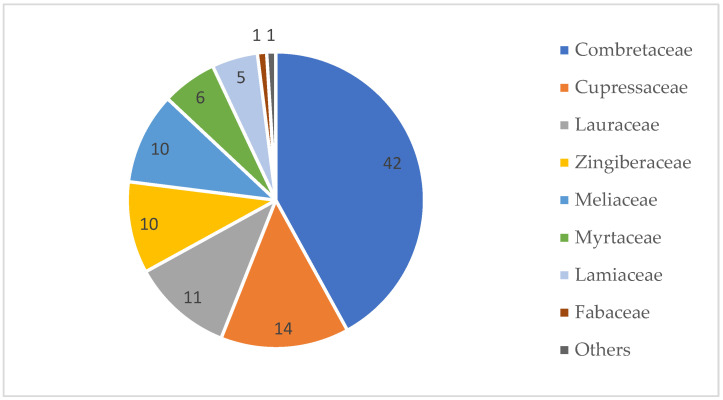
The significant role of plants of the family Combretaceae among antibacterial plants of the world. The percentages indicate the percent of genera with antibacterial properties within a plant family. Modified from Prasad et al. [12].

This review compiles the botany, ethnopharmacology, and antimicrobial potential of some African species of the genera *Combretum* and *Pteleopsis*, both belonging to the pantropical Combretaceae family [12,13,14,15,16,17,18]. A study which aimed to reveal plant taxa with antimicrobial properties confirmed that the family Combretaceae demonstrated the largest relative number of genera and species with antimicrobial properties [12], Figure 1. This result encourages further studies on the antimicrobial potential of plant species belonging to this family. In Africa, many traditional medicinal uses of *Combretum* and *Pteleopsis* species are related to the treatment of infections and their symptoms [13,19,20,21]. Moreover, there are several herbal formulations of plant extracts from the *Pteleopsis* and *Combretum* species, in both African and international markets, that are briefly discussed in this review [20].

In accordance with their traditional uses for the treatment of infection, numerous in vitro studies have confirmed that African *Combretum* species possess antibacterial and antifungal properties [22,23]. Species of the genus *Pteleopsis* have been studied to a lesser extent, although there are reports confirming the antimicrobial activities of *P. myrtifolia* and *P. suberosa* [24,25]. Most of the studies report on the activities of extracts and, in some cases, active compounds have been isolated. Antimicrobial compounds in *Combretum* and *Pteleopsis* species include ellagitannins, ellagic acid derivatives, gallotannins, terpenoids, saponins, fatty acids, fatty alcohols, flavonoids, stilbenoids, lignans, non-protein amino acids, and alkaloids [26,27,28]. In this review paper, the antimicrobial effects of extracts from various parts and with various polarities from African *Combretum* and *Pteleopsis* spp. are summarized. In addition, a number of secondary metabolites isolated from the genera are discussed in relation to their antimicrobial activities.

Studies have shown that extracts and compounds from some species of *Combretum* have strong synergistic and/or additive effects when used with conventional antimicrobial agents and with other plant extracts [19,29,30,31]. In this context, it is important to note that there are no combination or synergistic studies on *Pteleopsis* species. The concept of using plant-derived extracts and compounds to increase and preserve the effects of conventional antibiotics is important when combating resistant bacteria and fungi. However, this aspect has not been studied enough with respect to the antibiotic adjuvant potential of extracts and compounds from the *Combretum* and *Pteleopsis* species. Altogether, this review presents some *Combretum* species that should be focused on in more detail regarding their potential as producers of antibiotic adjuvants.

Although a number of ethnopharmacological studies have been performed on *Combretum* and *Pteleopsis* species, there is still a vast amount of undocumented information; most of the knowledge on the traditional medicinal uses of these plants is passed down orally [32]. The loss of forests and other habitats, as well as the movement of young people to cities in Africa, has also accelerated the loss of information concerning medicinal plants in general [32]. Therefore, this review aims to compile the documented ethnopharmacological information in order to facilitate future ethnopharmacological research on species that have not been studied much in this respect. Moreover, extracts and compounds with confirmed antibacterial and/or antifungal activities—either alone or in combination studies with conventional antibiotics—are presented (preferably in relation to the ethnopharmacological use of the plant, if this information is available). The genera and species presented in this review contain valuable scaffold molecules for new antibiotics and/or antibiotic adjuvants. This review will serve as a reference guide and will provide insight for further studies that aim to find (new) antimicrobials and antibiotic adjuvants. It will also guide studies that aim to find antimicrobial extracts from the African *Combretum* and *Pteleopsis* species, both with respect to follow-up studies and to encourage the study of as-yet-unexplored species.

## 2. Methodology

The data sources used in the literature search were the Scopus, Elsevier, EBSCOhost, PubMed, Google Scholar, African Plant Database, The Plant List Database, and SciFinder electronic databases. The search phrases used included “ethnopharmacology and antibacterial activity of *Pteleopsis* species”, “ethnopharmacology and antibacterial activity of *Combretum* species’’, “antifungal activity of *Pteleopsis* species”, “antifungal activity of *Combretum* species”, “phytochemistry of *Pteleopsis* species’’, “phytochemistry of *Combretum* species”, “*Combretum* species and infections’’, “*Combretum* species and traditional medicine”, “*Pteleopsis* species and infections”, and “*Pteleopsis* species and traditional medicine”. Articles published between 1962 and 2022 were considered in the review. The older references were especially important regarding the ethnobotanical uses of the *Combretum* and *Pteleopsis* spp. in Africa. Many articles were accessed, and some inclusion and exclusion criteria were applied to include only the relevant articles. The CAS SciFinder^n^ 2022 and CAS registry number databases were used to identify and authenticate the detected chemical structures in the *Combretum* and *Pteleopsis* spp. ChemDraw^®^ software was used to draw the molecular structures in this review paper.

## 3. Botany, Ethnopharmacology, and Importance in Herbal Markets of African *Combretum* and *Pteleopsis* Species

### 3.1. The Genus Combretum

#### 3.1.1. Botany

**Figure 2:**
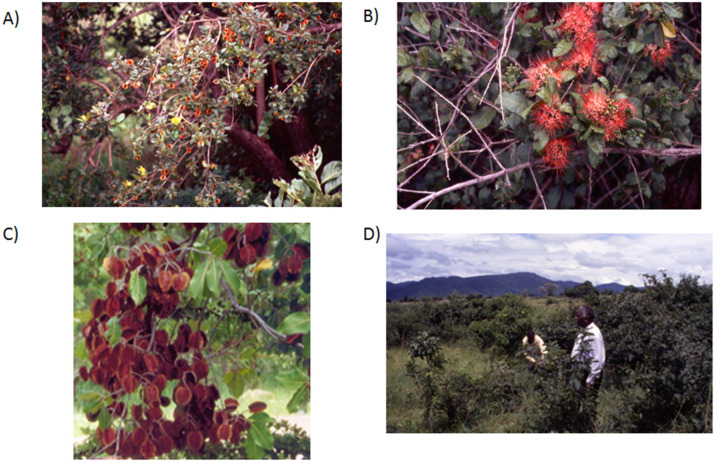
*Combretum* species in Africa. (**A**) *Combretum hereroense* in savanna woodland; (**B**) *Combretum constrictum* in riverine forest vegetation; (**C**) fruiting branch of *Combretum zeyheri*; and (**D**) *Combretum* orchard in Tanzania with *C. molle*. Photos: Pia Fyhrqvist.

Approximately 140 *Combretum* species occur in tropical Africa, including deciduous or semi-deciduous trees and shrubs as well as woody climbers and, in some rare cases, subshrubs with woody rootstocks (Figure 2) [16,33]. The flowers are inconspicuous or showy, with red, pink, yellow–green, yellow, or cream–white petals. The fruits, numbering between four and five, are winged with a thin, papery pericarp. The base of the leaf petioles may persist as spines, such as in *C. constrictum* (Figure 2B). The morphology of the fruit is considered useful for species identification. Moreover, the trichomes—glandular hairs on the leaves— have been found to be useful for the classification of *Combretum* species in combination with other taxonomical characters [16]. However, despite extensive anatomical and taxonomic research, the taxonomy and nomenclature within the genus *Combretum* is still unclear [16]. A large number of *Combretum* spp. occur in deciduous, savanna woodland habitats (such as the Miombo woodlands in East Africa) and wooded grasslands. Additionally, some species occur in rainforest habitats [34,35].

#### 3.1.2. Ethnopharmacology

At least twenty-four (24) species of *Combretum* are well-known in traditional African medicine, with a diverse range of uses that include remedies for snake and scorpion bites, worms, malaria, and topical and internal bacterial and fungal infections [36]. Sixteen of these *Combretum* species are discussed in this review. A number of *Combretum* species are traded in herbal markets in Limpopo, South Africa, Tanzania, and the Republic of Benin [37]. Moreover, some *Combretum* species such as *C. adenogonium* and *C. micranthum* are used by African immigrants in New York, U.S.A., and are therefore commonly available there in herbal medicine markets [38]. Traditional herbal remedies made from *Combretum* species include hot-water decoctions, cold-water extracts (macerations), infusions, teas, tinctures, porridges, and fresh leaf juices [29,36,37,39]. Volatile compounds in the plants may also be inhaled; for example, as a smoke inhalation of burnt plant material or as fumes from steam baths or hot water extracts. In addition, dried or fresh plant material is applied topically for wound care as ointments/dressings. All parts of the *Combretum* species—in some cases even the fruits or seeds—are used for medicinal purposes, although the fruits of some species are thought to be poisonous [40]. Table 1 summarizes some *Combretum* species used for the treatment of infectious diseases and their symptoms in African traditional medicine. The geographical occurrence, main botanical characters, and traditional medicinal uses of the species of *Combretum* in Table 1 are explained in more detail in the next paragraphs.

*C. molle* (the soft-leaved *Combretum*, velvet bush willow, and velvet-leaf willow) occurs in woodlands, open woodlands, rocky areas, and wooded grasslands throughout tropical Africa and in the Arabian Peninsula (Figure 2D). It grows into a medium-sized or large tree (up to 17 m) with a dense crown and rough, reticulate, and fissured bark [41]. The leaves are oppositely arranged, and the lamina is pubescent above and covered with dense, grey hairs on its lower surface. The greenish yellow, fragrant flowers are borne in axillary spikes. The elliptic- to oval-shaped fruit is a four-winged samara fruit with an apical peg [40]. *C. molle* is widely used in African traditional medicine for fever, colds, wounds, and pain. Moreover, the plant is used to treat a range of topical and internal infections, including HIV/AIDS [41]. Roots, leaves, and stem bark are the most commonly used parts, while the fruits are more seldom used [40]. The inner part of the root is used in wound dressings, and root decoctions are used for dysentery. The stem bark is used for the treatment of stomach problems. The leaves are used as anthelminthics and antidiarrheals [40]. In Mali, decoctions and powders are most commonly used in preparations for the treatment of dermatitis [42]. In Ethiopia, aqueous extracts of the stem bark are used for tuberculosis [43]. The seeds of this plant are widely used for the treatment of malaria by residents of rural areas of Ethiopia, mainly in the Gambella regional state [40].

*C. molle* can be found in herbal medicine markets in Africa. As this species is common in Africa, it is not on the list of vulnerable or endangered species, unlike many other medicinal plants. In Limpopo, South Africa, the roots are sold for the treatment of tuberculosis and skin disorders [37]. In Tanzania especially, the roots of *C. molle* are sold in marketplaces [44]. *C. molle* is also traded as an important medicinal plant in the Siby village in the Dioilan region of Mali, where the most common production methods for *C. molle* are decoctions and powders. In Mali, the most commonly used plant parts are leaves (37.7%), trunk bark (18.6%), whole plant (13.0%), and roots (10.7%) [42]. In addition to being an important ingredient as a single plant species in traditional medicine, *C. molle* is also commonly mixed with other plants: the roots of *C. molle* are mixed with the roots of *Annona chrysophylla* Boj. or *Annona senegalensis* Pers. to produce mucus-secreting or expectorant effects to treat coughs. In the treatment of syphilis, the roots of *C. molle* are combined with the following species, among others: *Securinega virosa* Pax et K. Hoffm. (Euphorbiaceae), *Psorospermum febrifugum* var. ferrugineum Keay & Milne-Redhead, and *Premna senensis* Klotsch (Verbenaceae) [17,36]. In the treatment of snake bites, the roots of *C. molle* are combined with the roots of *Markhamia obtusifolia* Sprague (Bignoniaceae) and *Vangueria rotundata* Robyn (Rubiaceae).

*C. micranthum* occurs in dry, often degraded savanna on stony and gravelly soils, outcrops, and termite hills, often following streambeds from sea level up to 1000 m altitude. *C. micranthum* (kinkéliba, synonym for medicine in some African languages) is an undomesticated shrub species found in the Tiger bush region (the name comes from tiger-patterned vegetation) of western Africa and in the Sahel region [45]. *C. micranthum* is a small tree (up to 10 m), shrub, or woody climber that can reach up to 20 m in height. It has pale grey bark and orange-to-red stems, with bark that peels in long, fibrous, red–brown strips [45]. Its leaves, which are hairy on the midrib, are oppositely arranged or are arranged in whorls of three, with a rounded base, brown scales beneath. The flower receptacle consists of two parts: the petals, which are free from each other and cream-coloured, and the style, which is up to 2.5 mm long and ranges from being hairy to almost glabrous. The flowers are typically rich in nectar and thus attract insects (especially bees), birds, and small mammals [45]. *C. micranthum* (Kinkéliba, bush tea) has been considered to belong to the fifty (50) most important medicinal plants in Africa and is inscribed in the French Pharmacopoeia [13,46]. It is used for the treatment of bruises and sprains by rubbing the root powder into shea butter or palm oil and applying the paste on the skin [45]. The roots are also used in decoctions as an anthelminthic to treat, e.g., guinea worm (*Dracunculus guinensis*) infestations, or as a wash in the treatment for open wounds [45]. *C. micranthum* leaves are used as an herbal infusion or tea, both of which are recognized to have therapeutic effects as antibacterial, antiviral, anti-inflammatory, and antimalaria remedies [47]. The most common use of kinkéliba is as a beverage made from the dried leaves, which is used for diuretic and digestion purposes, including gastrointestinal problems, colic, and vomiting. The dried leaves have been found to have hypoglycemic activity [20,27]. The leaves of *C. micranthum* (maceration/decoction/oral/bath) are used as an antimalarial medicine in malaria-endemic areas of the Ségou region, Mali [48,49]. In Guinea, *C. micranthum* is used for the treatment of tuberculosis [45]. In Senegal, Mali, Burkina Faso, and western Africa, *C. micranthum* is widely used as a general panacea [13]. The leaves, stem bark, and roots of *C. micranthum* are widely sold in local markets for a variety of diseases. In Mali, the powdered plant parts are sold in the form of tea bags to treat liver problems and diarrhea.

*C. imberbe* (Leadwood) occurs mainly south of the equator in open woodland and wooded savanna, growing in a wide variety of soils from sandy soils to limestone outcrops. It is most commonly found along rivers. *C. imberbe* is a deciduous shrub or small- to medium-sized tree (up to 20 m tall). The color of the trunk is pale grey or dark grey, and the snakeskin-like bark is its main morphological feature. The leathery leaves are arranged opposite to one another, hairless and grey–green [16]. The sweetly scented flowers are borne in axillary spikes and are yellowish cream colored [16]. Flowers are produced from November to March. The fruit is a four-winged samara, yellowish green with silvery scales. The colour of the mature fruits turn to pale red from February to June. *C. imberbe* is used throughout southern Africa as a traditional medicinal plant [50]. The powdered roots or leaves or their decoctions are used as a treatment for stomach problems and diarrhea, colds, coughs, and chest pains, all of which are symptoms that can be related to bacterial and fungal infections [51]. As a remedy for coughs, the smoke of burnt leaves is inhaled or the leaves are chewed [52]. General STI infections (sexually transmitted infections) are treated by crushing the leaves, suspending them in water, and drinking the remedy as an infusion [53,54,55]. In Namibia (Oshikoto region), *C. imberbe* infusions are used for two to seven days to cure gonorrhea [56]. In Zimbabwe, Zambia, Namibia, and Mozambique, *C. imberbe* is used for treating malaria, diarrhea, and bilharzia [57,58]. The root infusion is used for female infertility, and the bark powder is applied externally as a treatment against leprosy. Its ashes are used in commercially sold toothpaste [52]. The flowers are used to make a cough medicine that can be bought in local markets. *C. imberbe* is used with *Sclerocarya birrea* subsp. caffra, *Diospyros lycioides*, *Combretum erythrophyllum*, and other species to restore or revive fertility in women.

*C. erythrophyllum* (river bushwillow, bushwillow, river *Combretum*) occurs in riverine forests or savannas with sufficient groundwater in South Africa, Botswana, Mozambique, Namibia, and Zimbabwe [59]. *C. erythrophyllum* is a medium to large deciduous tree, growing up to 10–12 m tall, often with multiple stems and a spreading crown with some branches growing near the ground, thus giving it a willow-like appearance. Additionally, a large number of upright branches can sprout from the branches growing near the ground [60]. The bark is a greyish brown and flakes with age to expose grey patches. When mature, the leaves are dark green and shiny, whereas the young leaves are yellowish and shiny. The leaves turn red in the autumn, hence the name *erythrophyllum*. The flowers, which grow on dense axillary spikes, are greenish yellow and lightly scented with a sweet scent. The four-winged fruit (between 10 and 15 mm in length) changes color from green to yellow-brown when it ripens. The seeds, borne in the winged fruit capsules, are poisonous [61]. *C. erythrophyllum* is widely used in South African traditional medicine. Root, stem, and bark decoctions are used to treat leprosy and as a cure for coughs; a decoction is drunk three to four times per day [60]. Leaf infusions and roots are used to treat abdominal pains, while the dried, powdered gum is used for wounds [60]. Fresh or powdered leaves and roots are inserted into the vagina as a cure and prophylactic for venereal diseases [60]. Leaves are used for coughs, colds, infertility, venereal diseases, diarrhea and dysentery, sores, and wounds [29]. In Zimbabwe and among the Zulu in South Africa, *C. erythrophyllum* is used to treat infertility, for the maintenance of pregnancy (stem bark), and to facilitate birth (seeds/fruits).

*C. aculeatum* is native to the Sudano-Sahelian savanna region and the forest–savanna mosaic regions in Africa. Its geographical occurrence extends in a belt from West to East Africa. *C. aculeatum* grows as a scandent shrub or woody climber (between 4.5 and 8 m tall) and the leaf petioles become spines when mature. The young branches are pubescent or pilose. The leaves are lightly pubescent on both sides of the lamina. The flowers are white and scented. The fruit is indehiscent, obovate, five-winged, and has a shiny, purplish color when young [62]. *C. aculeatum* is a popular medicinal plant in the Sudano-Sahelian area [63]. In Sudan, extracts of the bark, seeds, and leaves are used to treat tuberculosis of the skin [63]. Additionally, extracts of the leaves are used as laxatives and to treat venereal diseases, extracts of the stem are used to treat skin infections such as leprosy, and decoctions of the root are used orally to treat influenza in Sudan [64]. Moreover, in Senegal, water decoctions of the aerial parts and root are used for TB and catarrh [65,66]. In Ethiopia, extracts of branches are used to treat eye problems and dysentery [63]. In Senegal, the Serer treat eye problems with the sap taken from the center of the stem. In Senegal, root powder is rubbed over the body to treat leprosy [66].

*C. adenogonium* (syn. *C. fragrans*, four-leaved bushwillow, four-leaved *Combretum*) occurs widely in tropical Africa, from Senegal and Guinea in West Africa to Ethiopia, Eritrea, Kenya, and Tanzania in East Africa. It is also found in Zimbabwe and Mosambique. Its common growth habitats are deciduous woodlands and wooded grasslands, often in association with seasonally waterlogged clay soils. *C. adenogonium* grows into a small tree (12–15 m tall) with reticulately fissured, grey bark and red twigs. The leaves are arranged oppositely or in whorls of three to four and are broadly to narrowly elliptic. The scented flowers are a greenish yellow, arranged in axillary spikes. The fruit is an almost-circular to elliptic four-winged nut (Samara fruit) with a yellow–brown to brown color [67]. *C. adenogonium* has numerous uses in African traditional medicine. Leprosy, coughs, diarrhea, and syphilis are treated with root decoctions of this plant [36,65,66]. In addition, leaf decoctions are used to clean wounds and for fungal infections of the scalp [36,44]. Moreover, decoctions of the stem, root, and leaves are applied to new and chronic wounds [68].

*C. apiculatum* (red bushwillow, rooibos—not to be confused with *Apalathus linearis*) is native to tropical eastern and southern Africa in woodlands, wooded grasslands, and *Acacia*-*Commiphora* shrublands. It grows into a small, much-branched, deciduous tree (up to 10 m tall). The leaves are oppositely arranged and shortly petiolate, and the leaf tip is apiculate. The yellow flowers are without fragrance and arranged in axillary spikes. The four-winged fruit has a yellowish green to reddish color [67]. Decoctions of the root are used for the treatment of leprosy and bloody diarrhea [65,69].

**Table 1:** Species of *Combretum* and *Pteleopsis* used in African traditional medicine for the treatment of infections and their symptoms.

Species Name and Geographical Occurence	Part of Plant Used and Herbal Preparation	Traditional Medicinal Uses	References
*C. aculeatum* Vent. From West to East Africa via the Sudano-Sahelian belt	Water decoctions of aerial parts and roots	Tuberculosis, laxatives, venereal diseases, leprosy, skin infections, colic, diarrhea, intestinal disorders, wounds, gastritis, eye treatments, stomach troubles	[63,64,65,66]
*C. adenogonium* Steud ex A. Rich. syn. *C. fragrans* F. Hoffm Widely distributed in tropical Africa from West to East Africa and south to Zimbabwe and Mozambique	Leaves, barks, and roots are used as decoctions, infusions and macerates	Diarrhea, leprosy, syphilitic sores, coughs, snakebites, wounds, sores, chest and abdominal pains, schistosomiasis, and fungal infections of the scalp	[17,36,44,65,66,67, 68]
*C. apiculatum* Sond. East, south, and southwestern Africa	Leaf extracts, leaf decoctions, and root decoctions	Stomach problems, disinfection of the navel after birth, venereal diseases, conjunctivitis, schistosomiasis, abdominal disorders, leprosy, and conjunctivitis	[66,69,70]
*C. collinum* Fresen. Widespread in dry savanna areas in tropical Africa. Occurs from Senegal to East Africa and south throughout southern Africa	Roots, boiled roots, barks, leaves, gum are used as decoctions; roots and twigs are chewed	Stomachache, purgative, diuretic, coughs, toothache, dysentery, snake bites, colds, chronic diarrhea, panaritium (nail bed inflammation), infertility, venereal diseases, sores, wounds, and malaria	[29,49,53,54,56,69,71]
*C. erythrophyllum* (Burch.) Sond. Native to southern African countries	Root, stem, and bark decoctions; dried powdered gum and leaves	Coughs, colds, leprosy, wounds and sores, prophylactic for venereal diseases, infertility, diarrhea, and dysentery	[29,35,60,61]
*C. hartmannianum* Schweinf. Horn of Africa, Sudan, South Sudan, Eritrea, Ethiopia	Roots, leaves, stem bark, stem wood, macerations, decoctions, tonics, pastes, ointments, and smoke fumigant	Abdominal pain, sore throat, dysentery, fever, jaundice, sexually transmitted diseases, fungal nail infections, rheumatism, fatigue, skin diseases, acne, wounds, ulcer infections, leprosy, and bacterial infections	[64,72,73,74,75,76,77,78]
*C. hereroense* Shinz In tropical Africa from Angola in West Africa to the Sudan in East Africa, as well as growing on a strip from Kenya to Zimbabwe	Shrub, leaves, and crushed leaves are suspended in water and used as a cold infusion Roots, leaves, young shoots, and barks are used as decoctions	Headache, female infertility, gonorrea, chlamydia symptoms in men, coughs, stomach problems, chest problems, schistosomiasis, abdominal ulcers, wounds, malaria, leprosy, and toncillitis	[35,51,53,54,69,71,79]
*C. imberbe* Wawra Occurs mainly in African countries south of the equator	Powdered roots, leaves or bark are used as decoctions; the smoke of burnt leaves is inhaled; leaves are chewed; infusions of leaves and roots are taken orally; and ashes of the wood are used as toothpaste	Stomach problems and diarrhea, colds, coughs and chest pains, sexually transmitted infections, malaria, bilharzia, female infertility, leprosy, viral, bacterial and fungal infections, and toothpaste	[51,52,53,54,55,56,57,58,80]
*C. kraussii* Hochst syn. *C. nelsonii* Duemmer	Leaf extracts, roots, and leaves	Bacterial respiratory diseases and wound healing	[22,81,82,83]
*C. micranthum* G. Don West African savanna region	Leaves, seeds, stem bark and roots are used as dried powders and decoctions, juice is made from fresh roots, root powder, and fruit (dried and fresh), steam baths, and infusions or tea	Wounds, burns, insect stings, nausea, coughs, bronchitis, fever, toothache, malaria, massage, sores, diuretic, diarrhea, ointment, treatment of bruises, colds, vomiting, and gastrointestinal problems	[20,45,47,48,49,53,54,84,85]
*C. molle* R. Br. ex G. Don Throughout tropical Africa and the Arabian Peninsula	Barks, roots, leaves, infusions, and twigs	Dental caries and bad smell, wound dressing, skin disorders, dysentery, snakebite, coughs, pneumonia, fever, inhalant for chest complaints, tuberculosis, leprosy, dysentery, stomach problems, edema, worms, gonorrhea, syphilis, venereal diseases, malaria and HIV, extracts of leaves inhaled as steam bath, and peeled twigs as chewing sticks	[13,35,36,37,40,41,42,43,59,69,70,86]
*C. nigricans* Lepr. ex Guill. et Perr. Sénégal, Mauretania, Niger, Burkina Faso	The gum exudated from the bark and roots	Gastrointestinal disorders, enteralgia (colic), stomach problems, acne, jaundice, arthritis, rheumatism, cataract, conjunctivitis, headaches, and malaria	[64,80,87,88]
*C. padoides* Engl. & Diels Tropical and south-eastern Africa	Leaves, roots, crushed leaves, decoctions, and water extracts	Snakebites, wounds, hookworms, malaria, diarrhea, conjuctivitis, and bacterial and fungal infections	[69,80,88,89]
*C. pentagonum* M. A. Lawson syn. *C. lasiopetalum* Engl. & Diels South-East Kenya to South Tropical Africa	Roots, leaves	Wounds, edema, gonorrhea, loose tooth, and bleeding gums	[71]
*C. psidioides* Welw Angola, Namibia, Tanzania, Zimbabwe	Decoction of roots; fresh, pounded leaves mixed with porridge; and in combination with *C. molle* and *C. zeyheri*	Diarrhea, oedema, and back and muscle pains	[36]
*C. zeyheri* Sond. From Kenya to eastern DR Congo and northern Namibia to north-eastern South Africa	Barks, roots, leaves, the smoke of burnt leaves, decoctions, water extracts	Smallpox, nose bleeding, hemorrhoids, diarrhea, bloody diarrhea, coughs, toothaches, bacterial and fungal infections, scorpion bite, dry wounds, schistosomiasis, and eye inflammation	[17,35,51,59,69]
*P. hylodendron* Mildbr. West and Central Africa, Cameroon	Decoctions of stem bark, leaf sap	Measles, chickenpox, sexually transmitted diseases, female sterility, liver and kidney disorders, and epilepsy	[90,91]
*P. myrtifolia* (Laws.) Engl. & Diels Kenya, Tanzania, Malawi, Zambia, Angola, Botswana, Zimbabwe, Mozambique and South Africa	Root, stem bark and leaves are used as decoctions, macerations and baths, leaf sap, soup of roots cooked with chicken, leaf sap mixed with leaf sap of *Diospyros zombensis* (B.L. Burtt) F. White, leaves and fruits as vegetables	Venereal diseases, sores, wounds, dysentery, menorrhagia, swellings of the stomach, wounds, muscle pain, and diarrhea	[17,92,93,94]
*P. suberosa* Engl. & Diels West Africa; Mali, Senegal, Guinea, Ghana, Togo, Benin, and Nigeria	Leaves, leafy twig infusions, root decoctions, roasted pulverized root is used topically for headache, extracts of chopped roots and young shoots, stem bark, and young branches are used as chew sticks Called “Terenifu” in Malian traditional medicine	Meningitis, convulsive fever, headache, jaundice, dysentery, dermatitis, stomachache, gastric ulcers, purgative, toothache, hemorrhoids, conjunctivitis, trachoma, gastrich ulcers, cataract, cough medicine, sexually transmitted diseases, hemorrhoids, viral diseases, and candidiasis	[24,95,96,97,98,99,100,101,102,103,104]

Abbreviations: HIV—Human Immunodeficiency Virus; DR Congo—Democratic Republic of Congo.

The leaves of *C. apiculatum* are used for the disinfection of the navel after childbirth, and decoctions of the leaves are taken in combination with steam bath treatments for stomach problems [70]. The stem bark is used for conjunctivitis [35].

*C. collinum* (Variable bushwillow) has a wide geographical occurrence in tropical and subtropical Africa [67]. It is a small, semi-evergreen tree or coppicing shrub [86] with a very variable morphology; several subspecies have been distinguished [72]. The bark is reddish brown to pale yellow, and the leaves are very variable in size, reaching sizes of up to 22 cm in length and 8 cm in width. The fragrant, yellow, cream, or white flowers are arranged in spikes; the winged fruit is a reddish brown to dark brown with a metallic appearance [67]. *C. collinum* is used together with *Combretum molle* and *Phyllanthus reticulatus* (Euphorbiaceae) to treat diarrhea [71]. The roots are made into decoctions for the treatment of dysentery [69]. Decoctions of the leaves are used to treat chronic diarrhea [53].

*C. hartmannianum* has a geographical occurrence restricted mainly to the Horn of Africa (Sudan, Ethiopia, Eritrea, and South Sudan) [73,105]. It grows into a shrub or small tree in the savanna woodlands, high-rainfall savannas, and wooded grasslands. The crown is broad and dense, and the shape of the leaves are characteristic for this species, having extremely extended tips [106]. The stem bark, roots, and leaves of this plant are used to treat jaundice [74]. In Sudan, the leaves are used as an ingredient in a medication used for jaundice, and smoke from the wood and bark is used to treat rheumatoid arthritis and dry skin [64,75]. Moreover, in Sudan, decoctions, macerations, and ethanolic tonics of the root and stem wood are used to treat a persistent cough, a symptom that could be related to tuberculosis [76]. In addition, *C. hartmannianum* is reported to be used for the treatment of fever and bacterial infections in Sudanese traditional medicine [77].

*C. hereroense* (russet bushwillow) is native to Angola, Botswana, Caprivi Strip, Ethiopia, Kenya, KwaZulu-Natal, Malawi, Mozambique, Namibia, Northern Provinces, Somalia, Sudan, Eswatini, Tanzania, Uganda, Zambia, and Zimbabwe. *C. hereroense* occurs in wooded grasslands (Figure 2A) and in *Acacia*-*Commiphora* bushlands. It is a small tree (8–12 m tall) or coppicing shrub. The leaves tend to cluster towards the ends of the twigs. The inflorescences (axillary spikes) often appear on leafless shoots, and the flowers are pale yellow to yellow and fragrant. The four-winged samara fruit is dark reddish to brown [67]. Decoctions of the root and young stem are taken as an oral medication in Namibian traditional medicine to treat tuberculosis, coughs, gonorrhea, and diarrhea [54]. In Zimbabwe, *C. hereroense* is used for the treatment of bilharzia [51]. Root decoctions are used for schistosomiasis and leprosy [69], and the shoots are used for toncillitis [79].

*C. kraussii* (syn. *C. nelsonii* Duemmer, *C. woodii* Duemmer, forest bushwillow) is native to southern Africa, where it occurs in the Cape Provinces, KwaZulu-Natal, Mozambique, Northern Provinces, and Eswatini. It grows as a shrub or small tree in forests and forest margins. The leaves are bright red in the winter and are narrowly to broadly elliptic with an entire but wavy margin. The leaf lamina can be up to 9 cm long. In association with the inflorscences, there is often a flush of new, whitish, smaller leaves. The greenish to creamy white flowers can number up to fifty in dense axillary heads. The fruit is a four-winged samara with a yellowish color and dark red wings [86]. The leaves of *C. kraussii* are applied to wounds, and leaf extracts are used for the treatment of respiratory diseases [22,81,82].

*C. nigricans* occurs from west tropical Africa to Ethiopia, where it grows in savanna regions and forest fringes. It is a small tree, growing up to 10 m. The bole is often twisted, and the bark is smooth. Two varieties occur: var. nigricans, with pubescent, leafy stems; and var. elliottii, with glabrous, leafy stems. During the hot season, the bark yields a gum, known as chiriri in Hausa, which is traded in the Sudano-Guinean region [66,87]. In Senegal, the bark and leaves are used as a cough medicine and expectorant [66]. An aqueous macerate is taken for colic and intestinal problems. The gum exudate from the stem is used for intestinal disorders, acne, jaundice, and rheumatism [64]. In Nigeria, the leaves are used to treat malaria [88].

*C. padoides* (thicket bushwillow) grows in the lowland areas of tropical and south-eastern Africa. It occurs in many habitats, from muddy riverbanks and dry woodlands to dry, rocky hillsides. Thicket bushwillow grows into a tree with drooping branches or a many-stemmed shrub. The leaves are arranged oppositely to suboppositely and have an acuminate apex. The flowers are a white to yellowish color and are arranged in spikes that can be up to 10 cm long. The four-winged fruits have a circular shape [67]. The name “padoides” comes from its resemblance to *Padus* spp. (Rosaceae). In traditional medicine, the leaves and roots are made into decoctions and cold-water extracts or the crushed leaves are used for bloody diarrhea, wounds, conjunctivitis, and malaria [80,88,89].

*C. pentagonum* is a liana or tree that grows in the seasonally dry tropical biome in eastern and southern tropical Africa. Root decoctions are used for hernia, hookworms, and dropsy. Root decoctions are mixed with porridge for the treatment of gonorrhea. In addition, root decoctions are used as a mouth rinse to treat bleeding gums and loosening teeth. Leaf decoctions are mixed with porridge for the treatment of gonorrhea [71].

*C. psidioides* (Peeling twig *Combretum*) grows to a tree (up to 17 m tall) or a large shrub in woodlands with sandy soils. The branchlets are usually tomentose when young, with bark that peels off in long, grey to black–purple strips, leaving a cinnamon-colored surface. The leaves are large, obovate, soft, oppositely arranged, and have a lower surface covered with dense hairs. In Tanzania, decoctions of the root of *C. psidioides* are used to treat diarrhea and muscle pain. The leaves are pounded and mixed with a maize porridge called Ugali to treat edema. In addition, *C. psidioides* is used in combination with *C. molle* and *C. zeyheri* to treat chest problems, pains in the spinal cord, and oedema [36].

*C. zeyheri* (large-fruited bushwillow) is a smallto medium-sized deciduous tree with a rounded crown. It occurs from Kenya to the DR Congo and the south to northeastern parts of South Africa in dry forests, savanna woodlands (*Brachystegia* woodlands), wooded grasslands, riverbanks, and dunes, especially in sandy soils. It also often grows on termite mounds. The size of the leaves and fruits of *C. zeyheri* is very variable. The branches are light brown and hairy. The leaves are oppositely arranged or in whorls of three. The flowers are greenish yellow and are arranged on spikes which can grow up to 8 cm long. The fruits are large and almost circular, four-winged, with a light brown color (Figure 2C). *C. zeyheri* has many uses in traditional medicine. The smoke of the leaves is inhaled to treat coughs. Water extracts of the leaves are used to treat colic. In Zambia, the leaves and stem bark are mixed with the roots of cassava to treat smallpox [59]. Decoctions of the leaves are used to treat eye inflammations (conjunctivitis). In addition, the leaves are pounded and mixed with oil for the treatment of back pain. Infusions and hot-water extracts of the roots are mixed with porridge for the treatment of diarrhea, dysentery, and vomiting [35]. *C. zeyheri* was one of the most popular medicinal plants among traditional healers in the Mbeya region, Tanzania, where decoctions of the leaves or roots are used as such or mixed with porridge to treat diarrhea [36]. Moreover, traditional healers in Mbeya sometimes mix *C. zeyheri* with other species of *Combretum* for the treatment of diarrhea [36].

### 3.2. The Genus Pteleopsis

#### 3.2.1. Botany

**Figure 3:**
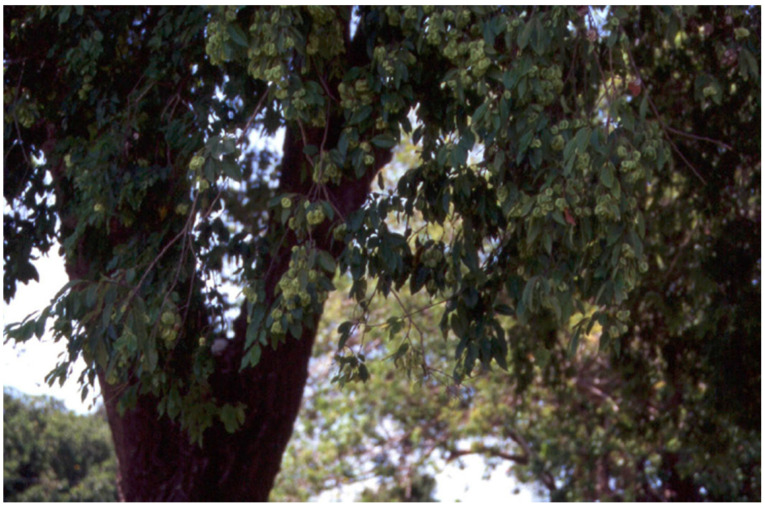
Fruiting *Pteleopsis myrtifolia*. Photo: Pia Fyhrqvist.

There are nine species of *Pteleopsis* in tropical Africa [66]. *Pteleopsis* species are small- to medium-sized trees or shrubs (Figure 3). In morphology, the genus is intermediatebetween *Combretum* and *Terminalia*. For example, the leaves lack scales or stalked glands, as in *Combretum* spp. The white-petaled flowers are arranged in terminal, axillary, or extra-axillary racemes, and hermaphrodite and male flowers are in the same inflorescence as in *Terminalia* spp. The fruits are two- to four-winged. *Pteleopsis* species occur in coastal bushlands, wooded grasslands, deciduous woodlands, riverine forests, and dry evergreen forests [67].

#### 3.2.2. Ethnopharmacology

Three species of *Pteleopsis* are used in African traditional medicine: *P. myrtifolia* in East Africa and *P. hylodendron* and *P. suberosa* in West Africa [46] (Table 1).

*Pteleopsis myrtifolia* (also known as stink-bushwillow or two-winged stinkbush) occurs in Kenya, Tanzania, Malawi, Zambia, Angola, Botswana, Zimbabwe, Mozambique, and South Africa. It is the only species of *Pteleopsis* that occurs in South Africa. Growth habitats of *P. myrtifolia* include evergreen and riverine forests and savanna woodlands. *P. myrtifolia* is a semi-deciduous, small tree with drooping branches, reaching heights up to 20 (–30) m (Figure 3) [66]. The bark is smooth, with a greyish pink color and a net-like appearance. The myrtle-like leaves are opposite and simple, with a glabrous–hairy and shiny lamina. The inflorescences are axillary to verticillate and contain both male and hermaphroditic flowers, white-petaled and strongly scented with an odor resembling honey or a strong smell. The fruit is yellowish green, turning brown when ripe, and papery thin with two to five wings [17]. A root decoction is taken to treat dysentery and stomachache, excessive menstruation, intestinal worms, and for fever [17]. Roots are boiled in water, and the decoction is drunk thrice a day for venereal diseases. It is applied externally to sores and wounds. Roots and leaf sap are used for the treatment of venereal diseases; the decoction is drunk against dysentery, menorrhagia, swellings of the stomach, and for treating wounds [17]. Roots, stem bark, and leaves are used for muscle pain and diarrhea as cold macerations, administered orally and as baths [92]. In Tanzania, the leaf sap of *P. myrtifolia* is drunk together with the leaf sap of *Diospyros zombensis* (B.L. Burtt) F. White (Ebenaceae) to treat dysentery. The roots are cooked with chicken, and the soup is taken to treat sterility. *P. myrtifolia* is also used as a medicine for female sterility [52]. In addition, *P. myrtifolia* is used for malaria in Mozambique, although the antimalarial properties are still unknown, and should thus be studied to validate this therapeutic use [92]. The leaves and fruits are considered edible, as a vegetable [93]. In Maputaland, a natural region in South Africa, the smoke of the wood is used to preserve food [94]. The leaves and roots of *P. myrtifolia* are only sold in local markets for medicine.

*P. suberosa* occurs in the savanna region of West Africa, and occurrences are recorded from Mali, Senegal, Guinea, Ghana, Togo, Benin, and Nigeria [95]. *P. suberosa* is a deciduous shrub or a small tree, growing between 6 and 10 m tall. The bark is distinctively covered with corky warts [96]. The leaves are sometimes alternate, slightly short-haired, and greyish green. The flowers are greenish yellow, while the fruits are winged and pale green, becoming brown at maturity. The leaves of *P. suberosa* are popularly known for the treatment of meningitis, convulsive fever, and headache; they are also used to treat jaundice and dysentery [95]. A decoction of the fresh roots is used as a medicine against dysentery, dermatitis, stomachache, and gastric ulcers, and as a purgative [24]. Infusions of the bark or the leafy twigs are taken to treat many diseases, such as jaundice, wounds, toothache, hemorrhoids, conjunctivitis, trachoma, and cataracts [97,98]. The roots, leaves, and stem barks of *P. suberosa* are used in the treatment of diabetes mellitus [99]. The roasted, pulverized root is rubbed on the head to treat headaches. An extract from the chopped roots and young shoots is taken as a cough medicine. Various parts of *P. suberosa* are used in traditional medicine throughout West Africa [96]. According to a survey on medicinal plants in the Ghanaian herbal markets, *P. suberosa* were the most frequently sold medicinal products [99]. The stem bark strips are sold with the medicinal indication, “to cleanse the uterus and to treat sexually transmitted diseases” [99]. In addition, the bark of *P. suberosa* is commonly used in Mali for the treatment of gastric ulcers [100]. Moreover, in the Malian folk medicine, the stem bark, commonly named ‘‘terenifu’’, is known as a traditional remedy against coughs, asthma, hemorrhoids, viral infections, and especially against ulcers. In Benin, the decoction of roots is used by traditional healers as a treatment for various diseases and conditions. The stem bark is used to treat dysentery, eruptive fever, and epilepsy [101,102]. *P. suberosa* is sold at the local traditional markets of southern Benin for the treatment of candidiasis [103], and for oral diseases in Burkina Faso [104]. Young branches are used as chew sticks.

*P. hylodendron* is a tree commonly found in the forest regions of West and Central Africa and in Cameroon. The tree can grow up to 25–40 m tall, occasionally reaching 50 m. It resembles—and may be confused—with *Terminalia ivorensis.* The aqueous decoction of the stem bark of *P. hylodendron* is used to treat measles, chickenpox, sexually transmitted diseases, female sterility, and liver and kidney disorders [90,91]. In Congo, the leaf sap is used as a wash to treat epilepsy.

## 4. Antibacterial and Antifungal Properties

A number of African *Combretum* species and some *Pteleopsis* species have been studied for their in vitro antibacterial and antifungal effects. The numbers of these studies has increased recently. The species of *Combretum* are better studied, whereas fewer studies are available on the *Pteleopsis* species. However, there are still many species that have not been studied in this regard. Several studies have mainly examined the effects of different extracts, while studies dealing with antimicrobial compounds from the species of *Combretum* and *Pteleopsis* are less common. Moreover, some antimicrobial screening studies cited in this review involved an ethnomedicinal component to facilitate the selection of plant species for the screenings and to verify the claimed folk-medicinal value of the plants to treat infectious diseases and other infections. However, there are still a large number of antimicrobial screenings of Combretaceae that did not include an ethnomedical selection of suitable plant species [22]. While the antimicrobial potency has mostly been assessed as the growth inhibitory effect, anti-biofilm effects were investigated in a few cases. A variety of techniques, such as agar diffusion, agar dilution, and broth dilution methods, have been used to detect antimicrobial activities. In addition, the microbial growth has been assessed using turbidity (optical density) or reagents that measure cellular respiration (such as tetrazolium salts and resazurin).

### 4.1. Antibacterial and Antifungal Effects of Combretum *spp.* Extracts

In Table 2, African species of *Combretum* that have been screened for their antibacterial and/or antifungal effects are summarized. The species were chosen for more in-depth discussions according to the number of studies referring to them in the ScienceDirect database. In some studies, species with a common occurrence in South Africa and with many uses in traditional medicine, including treating infections, have been screened for their antibacterial and antifungal properties [13,22,107,108]. Some studies have included a large number of species. Masoko et al. [108] screened twenty-four South African species for their antifungal effects, Eloff [107] screened twenty-one species for their antibacterial effects, and Anokwuru et al. [109] screened twenty-eight species of *Combretum* against a panel of human pathogenic bacteria. Most studies on the antibacterial effects of *Combretum* species on bacteria that cause respiratory diseases have used only one bacterium, either *Pseudomonas aeruginosa* or *Klebsiella pneumoniae*, although a multitude of bacterial strains, such as *Staphylococcus aureus*, *Streptococcus pneumoniae*, *Haemophilus influenza*, *Corynebacterium diphteriae*, *Bordetella pertussis,* and *Mycobacterium tuberculosis* are known to cause respiratory diseases [22]. Moreover, to date, *C. psidioides*, *C. padoides*, *C. zeyheri*, *C. hartmannianum*, *C. molle*, *C. apiculatum, C. imberbe*, and *C. hereroense* are the species that have so far been screened for their antimycobacterial effects, although there are still many *Combretum* species used for tuberculosis (TB) that have not been scientifically validated [23,51,107,110,111,112,113]. For example, *C. micranthum* is used for TB in Guinean traditional medicine, but there is no literature available about its antimycobacterial potential.

The antibacterial and antifungal potency of African *Combretum* species varies significantly between different species, plant parts, and extracts, as well as with the growth locality (Table 2). MIC values from 0.009 mg/mL up to 5 mg/mL, and sometimes even higher values (>6 mg/mL), are reported. However, in general, organic extracts of *Combretum* spp. have been reported to be more active than aqueous extracts [19]. Exceptions to this are known, such as a water extract of the leaves of *C. molle,* which inhibited the growth of *Fusarium* spp. (*F. proliferetum*, *F. solani*) with an MIC value of 40 µg/mL [19]. Moreover, extracts from a broad range of polarities have shown good antibacterial and antifungal activities; therefore, antimicrobial compounds in *Combretum* spp. are found both among non-polar, medium-polar, and polar compounds [28,114]. Regarding plant extracts in general, extracts demonstrating MIC values lower than 100 µg/mL are regarded as strongly active, while extracts possessing MIC values between 100 and 500 µg/mL are regarded active [115]. As can be seen from Table 2, extracts of several species of *Combretum* showed strong antibacterial and/or antifungal activity, with MIC values well below 100 µg/mL. In addition, numerous species have MIC values within the 100 to 500 µg/mL range [15,28]. In some cases, the *Combretum* species were selected for antibacterial or antifungal assays due to their uses for the treatment of topical or internal infections in African traditional medicine. In addition, species with no known (documented) ethnopharmacological uses have been screened. In this review, species of *Combretum* that are important in African traditional medicine, and which have many documented uses for the treatment of infections and their symptoms, are discussed in more detail to summarize the screening results on their in vitro antimicrobial effects.

**Table 2:** Antibacterial and antifungal effects of extracts of African *Combretum* species.

Plant Extracts	MIC/IZ/IZD	Reference
***C. acutifolium* Exell. (leaf)** Acetone, hexane, DCM, and methanol extracts	MIC range: 0.02–2.5 mg/mL against *C. albicans*, *C. neoformans*, *A. fumigatus*, *S. schenckii*, and *M. canis.*	[108]
***C. acutifolium* (leaf)** Methanol extract	MIC range: 0.15–1.50 mg/mL against *S. aureus*, *B. cereus*, *S. epidermidis*, *E. faecalis*, *E. coli*, *S. sonnei*, *S. typhimurium*, *P. aeruginosa*, and *K. pneumoniae.*	[109]
***C.adenogonium* Steud ex A.Rich syn. *C*. *fragrans* F. Hoffm. (leaf)** Water, methanol, and n-hexane	MIC values: 1 mg/mL (*B. cereus*, *K. pneumoniae*), 0.01562 mg/mL (*B. cereus*), and 0.25 mg/mL (*S. aureus*).	[116]
***C. fragrans* F. Hoffm. syn. *C. adenogonium* (leaf)** Ethanol extracts	MIC 0.25–4 mg/mL (*Candida* species) MIC between 0.5 and >4 mg/mL (Filamentous micromycetes).	[117]
***C. fragrans* F. Hoffm. syn. *C. adenogonium* (root)** Methanol extracts	IZD between 0 and 38 mm (Gram-positive and Gram-negative bacteria and *Candida albicans*). Best result: 38 mm against *Micrococcus luteus.*	[36]
***C. fragrans syn*. *C. adenogonium* (root)** Methanol extracts	Antifungal against *Candida albicans*, *C. krusei*, *C. glabrata*, *C. parapsilosis,* and *Cryptococcus neoformans*. Best result against *C. glabrata*: IZD 26 mm.	[17]
***C. adenogonium* (leaf)** Acetone extracts	MIC 0.625 mg/mL (*E. coli*)	[118]
***C. albopunctatum* Suess. (leaf)** Acetone and hexane extracts	MIC 0.08 mg/mL (*C. neoformans* and *A. fumigatus*).	[108]
***C. albopunctatum* (leaf)** Methanol extracts	MIC range: 0.75–3 mg/mL against *S. aureus*, *B. cereus*, *S. epidermidis*, *E. faecalis*, *E. coli*, *S. sonnei*, *S. typhimurium*, *P. aeruginosa*, and *K. pneumoniae.*	[109]
***C. albopunctatum* (leaf)** Acetone extracts	MIC values ranging between 0.02 and 0.64 mg/mL against *C. albicans*, *C. neoformans*, *M. canis*, *S. schenckii*, and *A. fumigatus.*	[83]
***C. albopunctatum* (leaf, stem bark)** Water extracts	Stem bark and leaf extracts inhibit the QS-dependent production of violacein and pyocyanin in *Chromobacterium violaceae* and *P. aeruginosa.*	[119]
***C. apiculatum* Sond. subsp. *apiculatum* (leaf)** Ethanol and water extracts	MIC values of ethanol extracts: 0.049 mg/mL against *B*. *subtilis* and *S. aureus.* MIC values of water extracts: 0.39 mg/mL against *B*. *subtilis* and *S. aureus.*	[120]
**C. *apiculatum* ssp. *apiculatum* (leaf)** DCM, methanol, and acetone	MIC 0.04 mg/mL (*C. albicans and C. neoformans*),	[108]
**C. *apiculatum* subsp. *apiculatum* (leaf)** Acetone extracts	MIC 1.6 mg/mL (*P. aeruginosa*), 0.4 mg/mL (*S. aureus*), 0.8 mg/mL (*E. coli*), and 0.8 mg/mL (*E. faecalis*).	[107]
***C. bracteosum* (Hochst.) Engl. & Diels (leaf)** DCM, methanol, and hexane	MIC 0.02 mg/mL (*C. neoformans*) MIC 0.02 mg/mL (*S. schenckii*)	[108]
***C. bracteosum*(Hochst.) Brandis (leaf)** Methanol extracts	MIC range: 0.50–3.00 mg/mL against *S. aureus*, *B. cereus*, *S. epidermidis*, *E. faecalis*, *E. coli*, *S. sonnei*, *S. typhimurium*, *P. aeruginosa*, and *K. pneumoniae.*	[109]
***C. caffrum* (Eckl. & Zeyh.) Kuntze (leaf)** Hexane and DCM extracts	MIC 0.16 mg/mL (*C. albicans*, *C. neoformans*).	[108]
***C. caffrum* (leaf)** Acetone extracts	MIC values: 6 mg/mL (*P. aeruginosa*), 0.8 mg/mL (*S. aureus*), 1.6 mg/mL (*E. coli*), and 0.4 mg/mL (*E. faecalis*).	[107]
***C. caffrum* (leaf)** Methanol extracts	MIC range: 0.63–2.50 mg/mL against *S. aureus*, *B. cereus*, *S. epidermidis*, *E. faecalis*, *E. coli*, *S. sonnei*, *S. typhimurium*, *P. aeruginosa*, and *K. pneumoniae.*	[109]
***C. celastroides ssp. celastroides* Welw. ex M.A. Lawson** **(leaf)** DCM, methanol, acetone, and hexane extracts	MIC range between 0.02 and >2.5 mg/mL *C. albicans, C. neoformans, A. fumigatus, Sporotrichum schenkii,* and *Microsporum canis.* Best results: DCM 0.08 mg/mL (*C. neoformans*); acetone and MeOH 0.02 mg/mL (*M. canis*).	[108]
***C. celastroides* ssp. *celastroides* (leaf)** Acetone extracts	MIC values: 3.0 mg/mL (*P. aeruginosa*), 0.8 mg/mL (*S. aureus*), 3.0 mg/mL (*E. coli*), and 1.6 mg/mL (*E. faecalis*).	[107]
***C. celastroides* ssp. *orientale* (leaf)** Acetone extracts	MIC values: 1.6 mg/mL (*P. aeruginosa*), 1.6 mg/mL (*S. aureus*), 3.0 mg/mL (*E. coli*), and 0.8 mg/mL (*E. faecalis*).	[107]
***C. celastroides* subsp. *celastroides* and *C. celastroides* subsp. *orientale* (leaf)** Methanol extracts	MIC range: 0.50–3.00 mg/mL and 0.25–3.00 mg/mL, respectively, against *S. aureus*, *B. cereus*, *S. epidermidis*, *E. faecalis*, *E. coli*, *S. sonnei*, *S. typhimurium*, *P. aeruginosa*, and *K. pneumoniae.*	[109]
***C. collinum* ssp. *Suluense* (Engl. & Diels) Okafor (leaf)** Acetone and DCM	MIC values: 0.08 mg/mL (*C. albicans* and *C. neoformans*).	[108]
***C. collinum* ssp. *Taborense* (Engl.) Okafor (leaf)** Acetone, DCM extracts	MIC values: 0.08 mg/mL (*C. neoformans*) and 0.64 mg/mL (*C. albicans*).	[108]
***C. collinum* Fresen. (leaf)** Acetone extracts	MIC values: 0.13 mg/mL (*S. aureus*), 0.07 mg/mL (*E. coli*), 0.08 mg/mL (*P. aeruginosa*), and 0.100 mg/mL (*E. faecalis*).	[121]
***C. collinum* (fruits, leaves, roots)** Methanol extracts	No activity against *Candida* spp. or *Cryptococcus neoformans*, with the exception of a leaf MeOH extract against *C. krusei* (IZD 18.4 mm).	[17]
***C. edwardsii* Exell (leaf)** Acetone and methanol extracts	MIC 0.04 mg/mL (*C. albicans*).	[108]
***C. edwardsii* (leaf)** Ethyl acetate fraction, DCM, hexane, and water fractions	MIC range from 0.390–3.125 mg/mL against *E. coli*, *K. pneumoniae,* and *S. aureus.*	[30]
***C. eleagnoides* Klotzsch (leaf)** Methanol extracts	MIC 0.05 mg/mL against *B. cereus* and a low average MIC value of 0.52 mg/mL against the other bacteria used in the screenings.	[109]
***C. erythrophyllum* (Burch.) Sond. (leaf)** Ethyl acetate and acetone extracts	MIC 0.04 mg/mL (*Fusarium* spp.).	[19]
***C. erythrophyllum* (leaf)** Methanol extracts	MIC 3.875 mg/mL (*C. albicans*, *A. niger*).	[29]
***C. erythrophyllum* (leaf)** Acetone, methanol, DCM extracts	MIC values: 0.02 mg/mL (*M. canis*), 0.32 mg/mL (*C. neoformans*, *S. schenckii*, and *M. canis*).	[108]
***C. erythrophyllum* (leaf)** Acetone extracts	MIC values: 3.0 mg/mL (*P. aeruginosa*), 0.8 mg/mL (*S. aureus*), 1.6 mg/mL (*E. coli*), and 1.6 mg/mL (*E. faecalis*).	[107]
***C. erythrophyllum* (leaf)** Methanol extracts	MIC range: 0.50–2.50 mg/mL against *S. aureus*, *B. cereus*, *S. epidermidis*, *E. faecalis*, *E. coli*, *S. sonnei*, *S. typhimurium*, *P. aeruginosa*, and *K. pneumoniae.*	[109]
***C. erythrophyllum* (leaf)** Water, CHCl_3_, butanol, 35% water in methanol, and CCl_4_ bioautography	MIC 0.05–25 mg/mL of solvent partition fractions against *S. aureus*, *P. aeruginosa*, *E. faecalis* and *E. coli* Best result for a 35% water extract in MeOH against *S. aureus* (MIC 0.05 mg/mL); a chloroform fraction contained the highest number of antibacterial compounds.	[114]
***C. hartmannianum* (Schweinf) (bark)** DCM, ethyl acetate, ethanol	MIC values of 12.5, 25 and 1.56 mg/mL, respectively, against *Mycobacterium aurum* A+.	[112]
***C. hartmannianum* (bark)** Methanol, 50% ethanol	MIC values of 0.5 and 1 mg/mL, respectively, against *Porphyromonas gingivalis.*	[122]
***C. hartmannianum* (fruit)** Water extracts	MIC 1.91 mg/mL, and IZD 20 and 19 mm against *B. subtilis* and *S. aureus*, respectively.	[74]
***C. hartmannianum* (leaf)** Methanol extracts	MIC 1.43 mg/mL, IZD 30 mm against *B. subtilis.*	[74]
***C. hartmannianum* (leaf)** DCM, ethyl acetate, ethanol	MIC values of 0.78, 3.12 and 0.19 mg/mL, respectively, against *Mycobacterium aurum* A+.	[112]
***C. hartmannianum* (root)** Ethanol extracts	MIC 0.2 mg/mL (*E. coli*, *S. aureus*).	[75]
***C. hartmannianum* (root)**	MIC 0.313 and 0.625 mg/mL, respectively, of a methanol and ethyl acetate extract of the root against *Mycobacterium smegmatis.*	[76]
***C. hereroense* Schinz (leaf)** Acetone, methanol, DCM and hexane extracts	MIC values of 0.02 mg/mL (*Cryptococcus neoformans*), 0.02–0.32 mg/mL (*Candida albicans*), and 0.02–0.04 mg/mL (*Microsporum canis*).	[108]
***C. hereroense* (leaf)** Hexane, DCM, acetone and methanol extracts	MIC values of 1.25, 0.62, 0.47 and 1.90 mg/mL, respectively, against *Mycobacterium smegmatis.*	[113]
***C. hereroense* (leaf)** Acetone extracts	MIC values: 1.6 mg/mL (*P. aeruginosa*), 3.0 mg/mL (*S. aureus*), 3.0 mg/mL (*E. coli*), and 1.6 mg/mL (*E. faecalis*).	[107]
***C. hereroense* (leaf)** Methanol extracts Water extracts	MIC values: 5.075 mg/mL (*A. niger*), 4.486 mg/mL (*C. albicans*), 0.395 mg/mL (*Rhizopus stolonifer*), 0.240 mg/mL (*Proteus vulgaris*), and 0.287 mg/mL (*Proteus vulgaris* and *Rhizopus stolonifer*).	[29]
***C. hereroense* (stem)** Methanol extracts	MIC values: 30.0 mg/mL (*S*. *epidermidis*) and 23.3 mg/mL (*Sarcina* sp.).	[36]
***C. imberbe* Wawra (leaf)** Hexane, DCM	MIC 0.16 mg/mL (*C. albicans and C. neoformans*).	[108]
***C. imberbe* (leaf)** Acetone extracts	MIC values: 2.5 mg/mL (*C. albicans*), 0.16 mg/mL (*C. neoformans*), 0.04 mg/mL (*M. canis*), and 2.5 mg/mL (*S. schenckii*, *A. fumigatus*).	[83]
***C. imberbe* (leaf)** Methanol extracts	MIC range: 0.05–0.75 mg/mL against *S. aureus*, *B. cereus*, *S. epidermidis*, *E. faecalis*, *E. coli*, *S. sonnei*, *S. typhimurium*, *P. aeruginosa*, and *K. pneumoniae.*	[109]
***C. imberbe* (leaf)** Acetone extracts	MIC values: 3.0 mg/mL (*P. aeruginosa*), 1.6 mg/mL (*S. aureus*), 3.0 mg/mL (*E. coli*), and 1.6 mg/mL (*E. faecalis*).	[107]
***C. imberbe* (leaf)** Ethanol extracts	MIC 0.125 mg/mL (*Mycobacterium smegmatis*).	[51]
***C. kraussii* Hochst (bark)** Ethyl acetate, ethanol, and aqueous extracts	MIC values between 0.6–9.0 mg/mLagainst *B. subtilis*, *S. aureus*, *E. coli,* and *K. pneumoniae.*	[123]
***C. kraussii* (leaf)** Hexane extracts	MIC 0.08 mg/mL (*C. albicans*)	[108]
***C. kraussii* (leaf)** Acetone extracts	MIC values: 1.6 mg/mL (*P. aeruginosa*), 0.8 mg/mL (*S. aureus*, *E. faecalis*), and 1.6 mg/mL (*E. coli*).	[107]
***C. kraussii* (leaf)** Ethyl acetate fraction, DCM, hexane fraction, and water fractions	MIC range from 0.390 to 1.560 mg/mL against *E. coli*, *K. pneumoniae,* and *S. aureus.*	[30]
***C. kraussii* (root)** Ethyl acetate, ethanol, and aqueous extracts	MIC values between 0.195–3.125 mg/mLagainst *B. subtilis*, *S. aureus*, *E. coli,* and *K. pneumoniae*.	[123]
***C. micranthum* G. Don (root, stem bark and leaves)** Water and methanol extracts	Agar diffusion IZD results: All root, bark, and stem bark extracts showed a strong growth inhibition of clinical isolates of *P. aeruginosa* at a level significantly higher than ampicillin, gentamycin, and ciprofloxacin. Hot-water extracts of the root bark inhibited the growth of clinical strains of *Streptococcus pyogenes*. The root and stem bark extracts were more active than extracts of the leaves.	[124]
***C. micranthum* (leaf)** Ethanol extracts	Active at 1 mg/mL and 5 mg/mL (*P. aeruginosa and S. aureus*) and at 5 mg/mL against *C. albicans* (IZ from 8 to 11 mm). MIC 0.5 mg/mL of an ethanol extract of the leaves against *S. aureus*.	[84]
***C. micranthum* (leaf)** Acetone extracts	MIC 310 µg/mL (*Mycoplasma mycoides* subsp. *mycoides*).	[125]
***C. micranthum* (stem bark)** A 70% EtOH extract and its solvent partition fractions; n-hexane, chloroform, and aqueous	70% EtOH extract and aqueous fraction: MIC 230 µg/mL (*E.coli*), MIC 470 µg/mL (*P. aeruginosa*), and MIC 940 µg/mL (*S. aureus*). n-hexane fraction: MIC 7.5 mg/mL (*S. aureus, E. coli*), and 15 mg/mL (*P. aeruginosa*). chloroform fraction: MIC 1880 µg/mL (*S. aureus, B. subtilis, and E. coli*).	[126]
***C. microphyllum* Klotzsch(leaf)** Acetone, methanol, DCM, and hexane extracts	MIC 0.02 mg/mL (*C. neoformans*)	[108]
***C. microphyllum* (leaf)** Acetone extracts	MIC values: 1.6 mg/mL (*P. aeruginosa*), 0.4 mg/mL (*S. aureus*), 0.8 mg/mL (*E. coli*), and 0.8 mg/mL (*E. faecalis*).	[107]
***C. microphyllum* (leaf)** Methanol extracts	3.9 mg/mL (*A. niger*), 1.008 mg/mL (*C. albicans*), and 0.494 mg/mL (*R. stolonifer*).	[29]
***C. microphyllum* (leaf)** Acetone and 1% aqueous sodium bicarbonate, hexane, ethyl ether, methylene dichloride, tetrahydrofuran, acetone, ethanol, ethyl acetate, methanol, and water.	MIC against *S. aureus*, *P. aeruginosa*, *E. coli* and *E. faecalis* varied from 0.08 to 1.20 mg/mL for the different extracts, with hexane providing the lowest MIC of 0.08 mg/mL against *E. faecalis*. The water extract was not as active as the other extracts (MIC 1.20 mg/mL).	[127]
***C. moggii* Excell (leaf)** Methanol extracts	MIC 0.02 mg/mL (*C. albicans and C. neoformans*).	[108]
***C. moggii* (leaf)** Acetone extracts	MIC 3.0 mg/mL (*P. aeruginosa*), 0.8 mg/mL (*S. aureus*), 1.6 mg/mL (*E. coli*), and 1.6 mg/mL (*E. faecalis*).	[107]
***C. molle* R. Br. ex. G. Don. (stem bark)** Acetone extracts	MIC 0.050 mg/mL (*Shigella* spp., *E. coli*)	[43]
***C. molle* (leaf)** Acetone extracts	MIC 0.625 mg/mL (*E. coli*)	[118]
***C. molle* (leaf)** Ethyl acetate and acetone extracts	MIC 0.04 mg/mL (*Fusarium* spp.)	[19]
***C. molle* (leaf)** Ethyl alcohol:H_2_O (50:50)	MIC 0.25 mg/mL (*Microsporum*, *Trichophyton*)	[46]
***C. molle* (leaf)** Acetone, methanol, DCM and hexane	MIC 0.02 mg/mL (*C. neoformans*)	[108]
***C. molle* (leaf)** Acetone extracts	MIC 0.160 mg/mL (*Mycoplasma mycoides* subsp. *mycoides*)	[125]
***C. molle* (leaf)** Methanol extracts	IZD 0–30 mm. Best results: 30 mm against *Micrococcus luteus,* 25 mm against *Enterobacter aerogenes,* and 25 mm against *Sarcina* sp.	[36]
***C. molle* (root)** Methanol extracts	MIC 1.00 mg/mL (*S*. *aureus*) A decoction was inactive.	[128]
***C. molle* (stem bark)** Ethanol extracts	MIC 0.250 mg/mL (*B*. *cereus*)	[129]
***C. molle* (stem bark)** Acetone extracts	MIC 1.000 mg/mL (*M. tuberculosis*)	[111]
***C. molle* (leaf)** Methanol extracts	MIC 0.040 mg/mL against *Penicillium janthinellum.*	[130]
***C. molle* (root)** Methanol extracts	Antifungal against all *Candida* spp. used in the screening and *Cryptococcus neoformans*. Best activity against *C. glabrata* (IZD 25.8 mm)	[17]
***C. mossambicense* (Klotzsch) (leaf)** Methanol and hexane extracts	Active against yeasts, dimorphic fungi and moulds at MIC values between 0.02 and 2.5 mg/mL. Lowest MIC values: 0.04 mg/mL of a methanol extract (*C. albicans*); 0.02 mg/mL of acetone, dichloromethane and hexane extracts (*M. canis*); and 0.02 mg/mL of a hexane extract (*C. neoformans*).	[108]
***C. mossambicense* (leaf)** Acetone extracts	MIC values: 0.800 mg/mL (*P. aeruginosa*, *S. aureus*), 1.600 mg/mL (*E. coli*), and 0.400 mg/mL (*E. faecalis*).	[107]
***C. nelsonii* Duemmer (Angustimarginata Engl. & Diels) syn. *C. kraussii* Hochst (leaf)** Hexane extracts	MIC 0.02 mg/mL (*C. neoformans*)	[108]
***C. nelsonii* (leaf)** Acetone extracts	MIC values between 0.02 and 0.16 mg/mL against *C. albicans*, *C. neoformans*, *M. canis*, *S. schenckii* and *A. fumigatus.*	[83]
***C. nelsonii* (leaf)** Acetone extracts	MIC values of 3.0 mg/mL (*P. aeruginosa*), 0.8 mg/mL (*S. aureus*), 1.6 mg/mL (*E. coli*) and 6.0 mg/mL (*E. faecalis*).	[107]
***C. nigricans* Lepr. (leaf)** Ethyl alcohol–water (50:50, *v*/*v*)	MIC values between 1 and >4 mg/mL against *C. albicans*, *Epidermophyton floccosum*, *Microsporum gypseum*, *Trichophyton mentagrophytes* and *Trichophyton rubrum.*	[46]
***C. nigricans* (entire root)** Ethyl alcohol–water (50:50, *v*/*v*)	MIC between 0.25 and >4 mg/mL against *C. albicans*, *Epidermophyton floccosum*, *Microsporum gypseum*, *Trichophyton mentagrophytes* and *Trichophyton rubrum.*	[46]
***C. padoides* Eng. & Diels (leaf)** DCM and acetone extracts	MIC 0.32 mg/mL (*C. albicans, C. neoformans)*	[108]
***C. padoides* (leaf)** 70% Acetone in acidified water (crude), water, hexane, ethyl acetate, and butanol fractions	MIC between 0.019 and 2.5 mg/mL against *C. albicans, C. neoformans, A. fumigatus, E. coli, E. faecalis, S. aureus,* and *P. aeruginosa.* MIC values of various extracts: Crude extract (70% acetone): 0.039 mg/mL (*C. neoformans*) Hexane fraction: 0.019 mg/mL (*E. coli, E. faecalis, S. aureus*) Ethyl acetate fraction: 0.019 mg/mL (*C. neoformans*) Butanol fraction: 0.019 mg/mL (*P. aeruginosa*)	[117]
***C. padoides* (leaf)** Acetone extracts	MIC values: 0.800 mg/mL (*P. aeruginosa, E. coli, and E. faecalis*), and 6.000 mg/mL (*S. aureus*).	[107]
***C. padoides* (root)** Methanol extracts	Antifungal against all *Candida* spp. and *Cryptococcus neoformans*. Best result against *C. glabrata*; IZD 29.1 mm MIC 6.25 mg/mL (*C. glabrata* and *Cryptococcus neoformans*)	[17]
***C. padoides* (stem bark)** Crude methanol extract and a butanol fraction resulting from solvent partition of the MeOH extract	Lowest MIC: 1250 µg/mL of a methanol extract MIC of a butanol fraction: 2.5 mg/mL Test bacterium: *Mycobacterium smegmatis*	[23]
***C. padoides* (stem bark, root)** Methanol	IZD range: 0–32 mm Best results: IZD 32 mm against *Enterobacter aerogenes* and 31 mm against *S. aureus*. Not active against *E. coli.*	[36]
***C. paniculatum* Vent. (leaf)** Acetone, methanol, DCM and hexane extracts	MIC 0.02 mg/mL (*C. neoformans*)	[108]
***C. paniculatum* (leaf)** Acetone extracts	MIC values: 1.6 mg/mL (*P. aeruginosa, S. aureus, and E. faecalis*), 0.8 mg/mL (*E. coli*).	[107]
***C. paniculatum* (root)** Methanol, water extracts	MIC values: 2.77 mg/mL (*S. epidermidis*), 1.85 mg/mL (*S. aureus*), and 14.44 mg/mL (*S. epidermidis, S. aureus*).	[128]
***C. pentagonum* Laws. (fruit)** Methanol extracts	MIC 3.44 mg/mL, IZD = 21 mm, (*B. subtilis*)MIC 6.87 mg/mL, IZD = 23 mm, (*S. aureus*)	[74]
***C. pentagonum* (bark)** Water extracts	MIC 4.86 mg/mL, IZD = 18 mm, (*B. subtilis*)	[74]
***C. petrophilum* Retief. (leaf)** Acetone, methanol, DCM, and hexane	MIC 0.02 mg/mL: acetone and methanol extracts against *C. albicans* and *M. canis*; acetone, hexane, dichloromethane, and methanol extracts against *C. neoformans.*	[108]
***C. petrophilum* (leaf)** Methanol extracts	MIC range: 0.50– >3.00 mg/mL against *S. aureus*, *B. cereus*, *S. epidermidis*, *E. faecalis*, *E. coli*, *S. sonnei*, *S. typhimurium*, *P. aeruginosa,* and *K. pneumoniae*	[109]
***C. psidioides* Welw. (stem bark and fruit)** Methanol extracts	IZD 16.0–24.6 mm against *C. krusei*, *C. glabrata*, *C. parapsilosis,* and *Cryptococcus neoformans*	[17]
***C. psidioides* (leaf)** Methanol extracts	IZD between 17–30 mm (diameter of hole: 12 mm) against *S. aureus*, *E. aerogenes*, *S. epidermidis*, *B. subtilis,* and *C. albicans*	[36]
***C. psidioides* (stem bark)** Methanol extract and its n-butanol and chloroform fractions resulting from solvent partition	IZD range: 14–29.00 mm, with the crude methanol extract being the most active (IZD 29 mm). Lowest MIC: 625 µg/mL of a methanol extract. MIC 2500 µg/mL for the n-butanol and chloroform fractions. Test bacterium: *M. smegmatis*	[23]
***C. woodii* Duemmer (leaf)** Hexane, DCM, methanol extracts	MIC values: 0.08 mg/mL (*C. albicans and C. neoformans*) and 0.02 mg/mL (*Microsporum canis*).	[108]
***C. woodii* (leaf)** Crude water and methanol extracts	MIC values: 0.078 mg/mL (*C. neoformans*), 1.250 mg/mL (*C. albicans*), 0.156 mg/mL (*E. faecalis*), 0.625 mg/mL (*E. coli, P. aeruginosa*), and 0.312 mg/mL (*S. aureus*).	[117]
***C. woodii* (leaf)** Methanol extracts	MIC range: 0.50–3.00 mg/mL against *S. aureus*, *B. cereus*, *S. epidermidis*, *E. faecalis*, *E. coli*, *S. sonnei*, *S. typhimurium*, *P. aeruginosa,* and *K. pneumoniae.*	[109]
***C. zeyheri* Sond. (leaf)** Methanol extracts	MIC range: 0.25–3.00 mg/mL against *S. aureus*, *B. cereus*, *S. epidermidis*, *E. faecalis*, *E. coli*, *S. sonnei*, *S. typhimurium*, *P. aeruginosa*, and *K. pneumoniae.*	[109]
***C. zeyheri* (entire plant)**	Active at 0.03 mg/mL against *C. albicans* and *Trichophyton mentagrophytes.* Screening with bioautography.	[131]
***C. zeyheri* (leaf)** Water and methanol extracts	MIC 6 mg/mL against *E. coli* and *B. subtilis.*	[132]
***C. zeyheri* (leaf)** Acetone and methanol extracts	MIC 0.02 mg/mL (*C. albicans*) MIC 0.08 mg/mL (*C. neoformans*)	[108]
***C. zeyheri* (leaf)** Acetone extracts	MIC values: 0.80 mg/mL (*P. aeruginosa and S. aureus*) and 1.60 mg/mL (*E. coli, E. faecalis*).	[107]
***C. zeyheri* (stem bark, fruits, root)** Methanol extracts	IZD between 0–33 mm. *Micrococcus luteus*: IZD was 33 mm for a stem bark methanol extract.	[36]

Abbreviations: IZD—inhibition zone diameter; IZ—inhibition zone; MIC—minimum inhibitory concentration, DCM—dichloromethane; MeOH—methanol, EtOH—ethanol; CHCl_3_—chloroform.

#### 4.1.1. *Combretum molle*

The wide use of *C. molle* in African traditional medicine for the treatment of topical and internal infections indicates that this plant contains antimicrobial compounds (Table 1). In accordance with its traditional uses, *C. molle* extracts are reported to possess antibacterial and antifungal activity against a large spectrum of bacterial and fungal strains [36]. The screening results from various authors are summarized in Table 2. Most in vitro studies have used the leaves and stem bark, while the roots are more seldom included, even though the roots have traditional medicinal applications as decoctions and ointments with antiseptic properties for the treatment of tuberculosis, skin diseases, and dysentery, amongst others [36,37,111]. Elegami et al. [74] have reported that fruit extracts have an antimicrobial activity, although the fruits of *Combretum* spp. are not usually recommended to be used for traditional medicine, since they are considered poisonous.

An acetone extract of the stem bark showed an MIC value of 50 µg/mL against *E. coli* and *Shigella* spp., which is the lowest reported MIC value of a *C. molle* extract against bacteria. [43]. Moreover, MIC values of 160–170 µg/mL were observed for acetone extracts of the leaves against *P. aeruginosa* and *S. aureus* [130]. Fyhrquist et al. [36] reported that methanol extracts of the leaves inhibited the growth of *E. aerogenes* and *S. aureus*. Ethanol extracts of the leaves and stem bark inhibited two clinical isolates of *S. aureus* and *Streptococcus agalactiae*, both of which can cause bovine mastitis [81]. Ethanol extracts of the stem bark of *C. molle* inhibited the growth of the food-pathogenic bacterium, *B. cereus*, with an MIC of 250 µg/mL [129]. Leaf extracts in acetone were mildly active against *E. coli*, with an MIC value of 625 µg/mL [118]. Moreover, mild growth-inhibitory effects were recorded for an acetone extract of the stem bark against *M. tuberculosis* ATCC 27294, with an MIC of 1000 µg/mL [111]. Additionally, a leaf extract inhibited *Mycoplasma mycoides* with an MIC of 160 µg/mL [125]. Compared to the stem bark and leaves, few screenings have used root material. For example, Steenkamp et al. [128] found that methanol extracts of the root inhibited the growth of *S. aureus* (MIC 1 mg/mL), whereas a hot-water extract (decoction) was inactive. These results support the uses of the *C. molle* leaf and stem bark extracts for the treatment of infectious diseases and their symptoms in traditional medicine. However, decoctions seem to be less effective when compared to ethanol extracts. Thus, ethanol could be used as an alternative extractant for traditional remedies of *C. molle*.

Several authors reported that extracts of *C. molle* inhibited the growth of both filamentous fungi and *Candida* species (Table 2). For example, Seepe et al. [19] found that the water, ethyl acetate, and acetone extracts of the leaves of *C. molle* displayed promising antifungal effects against *Fusarium solanii* and *F. proliferetum*, with an MIC value of 40 µg/mL for all extracts. It was also noted that these effects were far better than for amphotericin-B (MIC 370 µg/mL). The strong antifungal effect of the water extract is noteworthy; water extracts of medicinal plants also have uses for the prevention and treatment of crop diseases among African smallholder farmers, as water is a readily available resource and the leaves of *C. molle* are a sustainable and renewable source for antifungals [19]. Moreover, Mogashoa et al. [130] found that an acetone extract of the leaves inhibited the growth of the plant-pathogenic fungus *Penicillium janthinellum* with a MIC of 40 µg/mL. In addition, Masoko et al. [108] found that acetone–leaf extracts were very good growth inhibitors of *Cryptococcus neoformans*, with a MIC of 20 µg/mL. In their screening, Asres et al. [43] found that an acetone stem-bark extract of *C. molle* inhibited *C. albicans* (MIC 400 μg/mL), *Aspergillus terreus* (MIC 1200 µg/mL), and strains of *Penicillium* (MIC 1500 µg/mL against all strains). Fyhrquist et al. [17] found that a methanol extract of the root was active against all screened *Candida* spp. as well as *Cryptococcus neoformans*. Finally, ethyl alcohol: water extracts (50:50) of the leaves of *C. molle* demonstrated good activity against the dermatophytes, *Trichophyton mentagrophytes* and *Microsporum gypseum*, which cause diseases on human skin [46].

#### 4.1.2. *Combretum erythrophyllum*

Although *C. erythrophyllum* is popular in southern African traditional medicine and is used for many diseases and symptoms that could have a bacterial etiology, such as venereal diseases, fewer studies report on its in vitro antimicrobial effects when compared to *C. molle* (Table 2). Moreover, no studies exist so far on the antimycobacterial effects of *C. erythrophyllum*, although its stem bark, leaf, and root decoctions are used for coughs (Table 1).

Fresh leaf acetone extracts provided MIC values ranging between 0.8 and 3.0 mg/mL against *S. aureus*, *E. coli*, *P. aeruginosa*, and *Enterococcus faecalis* [107]. Fractions obtained from leaf material using solvent partition with CCl_4_, CHCl_3_, 35% water-in-MeOH, butanol, and water were tested for their antibacterial effects, with the 35% water in methanol extract showing the lowest MIC of 0.05 mg/mL against *S. aureus* [114]. Anokwuru et al. [109] found that leaf methanol extracts of *C. erythrophyllum* were active against *Salmonella typhimurium* and *B. cereus*, with MIC values ranging from 0.32 to 0.5 mg/mL. Martini et al. [60] isolated several antibacterial flavonoids from a leaf extract of *C. erythrohyllum*. Acetone, hexane, dichloromethane, and methanol extracts of the dried leaves of *C. erythrophyllum* were active against *Candida albicans*, *Cryptococcus neoformans*, *Aspergillus fumigatus*, *Sporothrix schenckii,* and *Microsporum canis*, with MIC values ranging between 0.02 and >2.5 mg/mL [108]. In agreement with this finding, Cock and Van Vuuren [29] also observed that methanol extracts of the leaves were active against *C. albicans* and *Aspergillus niger*. Additionally, Seepe et al. [19] found that ethyl acetate and acetone extracts of the leaves of *C. erythrophyllum* showed promising growth-inhibitory effects against plant-pathogenic *Fusarium* spp., with MIC values ranging from 0.04 to 0.08 mg/mL. In the same screening [19], water extracts also showed activity against *F. solani* and *F. proliferatum*, though with slightly higher MIC values (0.16 and 0.31 mg/mL, respectively) when compared to the ethyl acetate (EtOAc) and acetone extracts.

#### 4.1.3. *Combretum adenogonium*

*Combretum adenogonium* (syn. *C. fragrans*) has numerous uses in African traditional medicine as a remedy against coughs, dysentery, diarrhea, septic wounds, and fungal infections on the scalp [17,36,133] (Table 1). Accordingly, various authors have justified these traditional uses (Table 2). Maregesi et al. [116] found that n-hexane and methanol extracts of *C. adenogonium* exhibited strong antibacterial effects against *B. cereus* (MIC 15.62 µg/mL). Fyhrquist et al. [36] demonstrated that methanolic leaf and root extracts of *C. fragrans* (*C. adenogonium* syn. *C. fragrans*) were active against *S. aureus*, *E. aerogenes*, *S. epidermidis*, *B. subtilis*, *Micrococcus luteus*, *Sarcina* sp., and *C. albicans*, with IZD values for the leaves ranging between 18 and 34 mm and between 22 and 38 mm for the roots. Additionally, Batawila et al. [117] showed in their screening that ethanolic extracts of *C. fragrans* were active against ten *Candida* species, with MIC values between 0.25 and 4 mg/mL, and against ten filamentous fungi, with MIC values between 0.5 and >4 mg/mL.

#### 4.1.4. *Combretum hartmannianum*

In the Sahel belt and in Sudan, *C. hartmannianum* is commonly used for the treatment of sore throats, dysentery, fever, sexually transmitted diseases, fungal nail infections, skin diseases, acne, wounds, ulcer infections, and leprosy (Table 1). All parts of the plant are used, and common preparations include decoctions, macerations, tinctures, pastes, ointments, and teas. These traditional medicinal uses indicate that *C. hartmannianum* contains antibacterial and antifungal compounds. Accordingly, some research has been performed on the antimicrobial activity of extracts of various parts of *C. hartmannianum* (Table 2). For example, bark extracts of *Combretum hartmannianum* demonstrated antibacterial activity against *Porphyromonas gingivalis*, a bacterium that causes periodontal diseases. The methanol extract showed the best activity (MIC 0.5 mg/mL), whereas a 50% ethanol extract was less active [122]. Water and methanol extracts of the stem bark, fruits, and leaves were mildly active against *B. cereus* (with MIC values from 1.43 mg/mL to 4.19 mg/mL) and *S. aureus* (with MIC values from 1.91 mg/mL to 8.39 mg/mL) [74]. In addition, ethanol, ethyl acetate, and dichloromethane extracts of the root and leaf inhibited the growth of both Gram-positive and Gram-negative bacteria at MIC values ranging from 0.1 to 3.13 mg/mL. The best results were provided by a leaf dichloromethane extract (MIC < 0.1 mg/mL) and a root dichloromethane extract (MIC 0.1 mg/mL) against *B. subtilis*. Moreover, ethanolic root extracts of *C. hartmannianum* inhibited the growth of *E. coli* with an MIC value of 0.2 mg/mL [75]. Only two studies to date report on the antimycobacterial effects of *C. hartmannianum*. In a study by Eldeen and Van Staden [112], it was shown that leaf extracts in particular, but also bark and root extracts, possessed growth-inhibitory effects against *Mycobacterium aurum,* with a leaf ethanol extract demonstratinging the best effects (an MIC of 0.19 mg/mL) followed by a stembark dichloromethane extract (an MIC of 0.78 mg/mL). Moreover, Salih et al. [76] showed that methanol and ethylacetate extracts of the root are active against *M. smegmatis*.

#### 4.1.5. *Combretum zeyheri*

In accordance with the traditional medicinal uses of *C. zeyheri* throughout Africa for diarrhea, coughs, eyewashes, toothaches, and bacterial and fungal infections (Table 1), a number of authors have found that extracts of this plant possess in vitro antibacterial and antifungal effects (Table 2). However, the MIC values reported against bacteria are in general quite high, and better growth-inhibitory effects were reported against fungi. Methanolic extracts of the dried entire plant of *C. zeyheri* were active against *C. albicans* and *Trichophyton mentagrophytes* at a concentration of 0.03 mg/mL when screened using a bioautographic method [131]. Acetone, hexane, methylene dichloride, and methanolic extracts of the dried leaves showed antifungal activities against *Candida albicans*, *Cryptococcus neoformans*, and *Aspergillus fumigatus,* with MIC values between 0.02 and 2.5 mg/mL [108]. Accordingly, Fyhrquist et al. [17] found that methanolic root and stem bark extracts had antifungal activities, particularly against the *Candida* species used in their screening, such as *C. albicans*, *C. krusei*, and *C. tropicalis*, as well as against *Cryptococcus neoformans*. Mapfunde et al. [134] found that, at 200 µg/mL, various extracts of *C. zeyheri*, enriched in flavonoids, alkaloids, and saponins as well as an ethanol extract of the leaves, inhibited the growth of *C. albicans* by 48–87%. Moreover, an alkaloid-enriched extract was found to give the highest inhibition (87%), followed by the ethanol extract (76%). However, the highest concentration used in the screenings, 200 µg/mL, did not result in the MIC for any of the tested extracts.

Water and methanol extracts of the dried leaves were active against *E. coli* and *B. subtilis* only at high concentrations, with an MIC value of 6 mg/mL [132]. However, in the same investigation, Masengu et al. [132] found that extracts of *C. zeyheri* leaves possessed an inhibitory effect on rhodamine efflux. Therefore, they suggested that *C. zeyheri* contains efflux-pump inhibitors that potentiate the antibacterial effects of compounds present in medicinal plants that are often mixed with *C. zeyheri* for traditional remedies. Fyhrquist et al. [36] showed that methanolic fruit, root, and stem bark extracts of *C. zeyheri* demonstrated antibacterial activity against *S. aureus*, *Enterobacter aerogenes*, *S. epidermidis*, *B. subtilis*, *Micrococcus luteus*, *Sarcina* sp., and *C. albicans*, with IZD values between 15 and 33 mm. However, the MIC values were not investigated in this investigation. Fyhrquist et al. [23] showed that methanol and butanol extracts of the stem bark and butanol extracts of the root of *C. zeyheri* inhibited the growth of *Mycobacterium smegmatis*. Additionally, Nyambuya et al. [135] showed that an alkaloid-enriched extract of the leaves of *C. zeyheri* inhibited the growth of *M. smegmatis* with an MIC value of 125 µg/mL, and the growth-inhibitory effect was concentration- and time-dependent. The good antibacterial and antifungal results of the alkaloid-enriched extracts [134,135] warrant more in-depth research on the antimicrobial alkaloids in *C. zeyheri*. Moreover, compounds with efflux-pump inhibitory activity should be characterized.

#### 4.1.6. *Combretum micranthum*

*C. micranthum* is used traditionally for the treatment of a variety of infections and is believed to have antibacterial properties [13], Table 1. In accordance with its uses for fever, coughs, bronchitis, burns, and wounds, and as a general antibiotic [45,84,85], extracts of *C. micranthum* have shown antibacterial and antifungal effects. Aqueous and methanol extracts of the stem bark, leaves, and root bark were screened against 200 clinical isolates of nosocomial bacteria [124]. All the bark extracts showed a strong growth inhibition of *P. aeruginosa* at a level significantly higher than ampicillin, gentamycin, and ciprofloxacin. The screened *P. aeruginosa* isolates were susceptible to the hot-water extracts of the root bark and stem bark of *C. micranthum*. Additionally, the hot water extract of the root bark also significantly inhibited the growth of *Streptococcus pyogenes*.

Ethanolic extracts (70% ethanol) of the stem bark of *C. micranthum,* collected in Nigeria, showed antibacterial effects against *E. coli* and *P. aeruginosa,* with MIC values of 230 and 470 µg/mL, respectively, and the same MIC values were also demonstrated by the aqueous solvent partition fraction of the ethanolic extract [126]. In contrast, the n-hexane fraction showed antibacterial activity only at high concentrations, with MIC values ranging from 7.5 to 15 mg/mL, whereas a chloroform fraction showed an MIC value of 1880 µg/mL against *S. aureus*, *E. coli,* and *B. subtilis*.

The fresh leaf extract of *C. micranthum* was bactericidal against *Shigella dysenteriae*, *Salmonella parathyphi B*, and *Klebsiella ozaenae*. It was bacteriostatic against *Shigella flexneri*, *S. boydii*, *Salmonella typhi*, *Klebsiella pneumoniae*, and *S. aureus* [136], thus supporting its traditional uses for diarrhea. The minimum inhibitory concentration of the ethanol extract and the n-hexane, chloroform, ethyl acetate, n-butanol, and aqueous fractions of *C. micranthum* leaves ranged from 0.62 to 15 mg/mL against *S. aureus*, *P. aeruginosa*, *K. pneumoniae*, *Candida albicans*, and *Trichophyton rubrum* [137]. The most purified fractions of C. *micranthum* leaves showed growth-inhibitory activity against methicillin-resistant *Staphylococcus aureus* (MRSA), *Clostridium difficile*, *E. coli*, and *P. aeruginosa*, with MIC values of 625, 156, 1250, and 1250 µg/mL, respectively [138].

#### 4.1.7. South African and Sudano-Sahelian Species of *Combretum*

In addition to the species discussed in detail in the previous paragraphs, a number of other *Combretum* species with good antibacterial potential are listed in Table 2. Many of these species have a geographical occurrence in southern and South Africa or in the Sudano-Sahelian region.

Methanol, dichloromethane, and n- hexane extracts of *C. acutifolium* were antibacterial and antifungal [108,109]. Several authors have found that the leaf extracts of *C. albopunctatum* show antibacterial and antifungal potential [83,108,109,119]. Leaf extracts of *C. apiculatum* inhibited both bacterial and fungal growth [107,108,120]. Methanol and dichloromethane extracts of the leaves of *C. bracteosum* showed strong antifungal effects against *Sporothrix schenkii* and *Cryptococcus neoformans*, with an MIC value of 20 µg/mL [108,109]. *C. collinum* leaf extracts were antibacterial, demonstrating good activity against *E. coli* (with an MIC of 70 µg/mL), and antifungal effects [17,108,121]. Leaf dichloromethane extracts of *C. celastroides* ssp. *celastroides* demonstrated potent antifungal effects against *C. neoformans* (MIC 90 µg/mL) and *M. canis* (MIC 20 µg/mL) [108]. Leaf extracts of *C. celastroides* ssp. *celastroides* and *C. celastroides* ssp. *orientale* were antibacterial, with activity profiles that slightly varied between extracts [107]. *Combretum kraussii* (syn. *C. nelsonii*) bark and root extracts had antibacterial effects [123]. Leaf extracts of *C. kraussii* were antibacterial and antifungal, with an n-hexane extract being particularly active against *C. albicans* (MIC 80 µg/mL) [108]. The antimicrobial effects of *C. kraussii* justify the traditional medicinal uses of this species for the treatment of wounds and bacterial infections [30,107,108]. *C. microphyllum* leaf extracts were antifungal, with the lowest MIC of 20 µg/mL against *Cryptococcus neoformans* [29,108]. In addition, leaf extracts of *C. microphyllum* were active against *E. faecalis*, with a lowest MIC value of 80 µg/mL [107,127].

### 4.2. Antibacterial and Antifungal Effects of Pteleopsis Species

Studies on the antibacterial properties of African *Pteleopsis* species are shown in Table 3. Although only three species—*P. myrtifolia*, *P. hylodendron*, and *P. suberosa*—are used in African traditional medicine, some additional *Pteleopsis* spp., such as *P. habeensis*, have also been studied for their antimicrobial potential.

#### 4.2.1. *Pteleopsis hylodendron*

An ethyl acetate extract from the stem bark of *Pteleopsis hylodendron* growing in West and Central Africa showed antibacterial effects against *Salmonella typhi*, *Corynebacterium diptheriae*, *Klebsiella pneumoniae*, *Proteus mirabilis*, *P. aeruginosa*, *Streptococcus pyogenes*, and *Bacillus cereus* [90]. Additionally, a methanolic stem bark extract of *P. hylodendron* demonstrated antibacterial effects against *S. aureus* (IZD 20.00–25.00 mm) and showed antioxidant effects [139].

#### 4.2.2. *Pteleopsis habeensis*

A 70% methanol extract of the stem bark of the West African species *P. habeensis* was active against *E*. *coli* and methicillin-resistant *Staphylococcus aureus* (MRSA) [140].

#### 4.2.3. *Pteleopsis suberosa*

Methanol extracts of the West African species *Pteleopsis suberosa* were found to possess antimicrobial activity against some skin-infection-causing bacteria, such as *Staphylococcus aureus*, *Staphylococcus capitis, S. epidermidis*, *Staphylococcus saprophyticus*, *Bacillus subtilis*, *Pseudomonas aeruginosa*, and *Pseudomonas cepacia* [141]. The methanol extract and a decoction of the stem bark of *P. suberosa* inhibited the growth of *Helicobacter pylori* ATCC 43504 and five clinical isolates of this bacterium known to cause gastric ulcers [142]. This finding supports the traditional use of decoctions from *P. suberosa* to treat gastric ulcers. Moreover, the use of *P. suberosa* for the treatment of gastric ulcers could be supported by the finding that aqueous extracts of the bark of *P. suberosa* contain high quantities of triterpenoid saponins that protect the gastric mucosa against ethanol and indomethacin-induced gastric lesions [24]. The antifungal effects of ethyl alcohol–water (50:50, *v*/*v*) extracts of the stem bark were observed in vitro against *Candida albicans*, *Epidermophyton floccosum*, *Microsporum gypseum*, *Trichophyton mentagrophytes,* and *T. rubrum* [46].

#### 4.2.4. *Pteleopsis myrtifolia*

Decoctions of the root, leaves, and leaf sap of *P. myrtifolia* are used for the treatment of wounds and bacterial infections, including dysentery. Some in vitro antimicrobial screenings could justify these traditional uses. Interestingly, according to Anokwuru et al. [109], methanol extracts of the leaves of *P. myrtifolia* were especially active against bacteria related to food spoilage and food poisoning, such as *Bacillus cereus*, *Shigella sonnei*, and *Salmonella typhi* (MIC 750 µg/mL). This result could support the use of *P. myrtifolia* leaf decoctions for the treatment of dysentery. In addition, the leaf sap of *P. myrtifolia*, combined with the leaf sap of *Diospyros zombensis*, is used for the treatment of dysentery. Thus, this plant combination should also be tested for its antibacterial effect. A screening made by Fyhrquist et al. [17] demonstrated that a methanol extract of the root of *P. myrtifolia* inhibited the growth of all *Candida* spp. used in the study as well as *Cryptococcus neoformans*, demonstrating the highest activity against *C. glabrata*. This study indicated that *P. myrtifolia* contains antifungal compounds that should be studied in more detail.

**Table 3:** *Pteleopsis* extracts with antibacterial and antifungal properties.

Plant Extracts	MIC/IZ/IZD	Reference
***Pteleopsis habeensis* Aubrev ex Keay** **(stem bark)** Methanol extracts	MIC/IZD against *E. coli*: 1.562 mg/mL (no growth), IZD 18–25 mm (at 12.5–100 mg/mL). MIC/IZD against *S. aureus*:1.562 mg/mL (no growth), IZD 18–24 mm (at 12.5–100 mg/mL).	[140]
***Pteleopsis hylodendron* Mildbr.** **(stem bark)** Crude methanol extracts	MIC 0.781–12.5 mg/mL: *E. coli*, *P. aeruginosa*, *P. mirabilis*, *S. flexneri*, *S. paratyphi* A/B, and *S. typhi.* MIC 0.781–3.125 mg/mL: *E. faecalis*, *S. aureus.* IZD against Gram-negative bacteria: 0.00–22.00 mm IZD against Gram-positive bacteria: 10.87–25.00 mm IZD against *S. aureus* (most sensitive): 20.00–25.00 mm	[139]
***Pteleopsis hylodendron*** **(stem bark)** Ethyl acetate extract	Ethyl acetate extract of the stem bark active against *Salmonella typhi, Corynebacterium diptheriae*, *Klebsiella pneumoniae*, *Proteus mirabilis*, *P. aeruginosa, Streptococcus pyogenes,* and *Bacillus cereus.*	[90]
***Pteleopsis myrtifolia* (M.A. Laws.) Engl. & Diels.** **(roots)** Methanol extracts	IZD 21.2 mm against *C. glabrata* IZD 16.9–21.2 mm; *C. albicans*, *C. krusei*, *C. tropicalis*, *C. glabrata*, *C. parapsilosis*, and *C. neoformans.*	[17]
***Pteleopsis myrtifolia*** **(leaves)** Methanol extracts	Average MIC 1.85 mg/mL ± 0.88 mg/mL against both Gram-positive and Gram-negative bacteria; *S. aureus, B. cereus, S. epidermidis, E. faecalis, E. coli, S. sonnei, S. typhimurium, P. aeruginosa,* and *K. pneumoniae.*	[109]
***Pteleopsis suberosa* Engl. et Diels** **(stem bark)** Methanol extracts ad decoctions	MIC-values: 0.03125–0.250 mg/mL and 0.0625–0.500 mg/mL, respectively, against *Helicobacter pylori* (ATCC 43504), and five clinical isolates of *H. pylori.*	[142]
***Pteleopsis suberosa*** **(stem bark and shoots/twigs)** Ethyl alcohol–water (50:50, *v*/*v*)	MIC-values: 0.25–1 mg/mL (stem bark) and 0.25–2 mg/mL (shoot) against *Candida albicans, Epidermophyton floccosum, Microsporum gypseum, Trichophyton mentagrophytes,* and *Trichophyton rubrum.*	[46]
***Pteleopsis suberosa*** **(stem bark)** Methanol extracts	Antimicrobial activity against some microorganisms causing skin infections, such as *Staphylococcus aureus*, *Staphylococcus capitis, S. epidermidis*, *Staphylococcus saprophyticus*, *Bacillus subtilis*, *Pseudomonas aeruginosa*, and *Pseudomonas cepacia.*	[141]

Abbreviations: IZD—diameter of inhibition zone; MIC—minimum inhibitory concentration.

### 4.3. Antimicrobial Screenings Comparing Species Belonging to Two or More Genera of Combretaceae

Papers including two or more genera of Combretaceae which were screened for their antimicrobial effects are presented in Table 4. These studies allowed for a direct comparison of the antimicrobial activities between closely related genera. There is a need for more screenings using large panels of taxonomically related plant genera and species to directly compare the antimicrobial potentials of different taxa [29]. Although this review focuses mainly on the genera *Combretum* and *Pteleopsis*, the antibacterial activities of these genera are compared to the closely related genera *Terminalia* and *Quisqualis*, also members of the plant family *Combretaceae*. In many cases, African *Terminalia* species showed better average antimicrobial effects than *Combretum* species [17,29,36], but also the opposite was seen in some studies [109].

In their recent study, Anokwuru et al. [109] screened the methanol leaf extracts of fifty-one species belonging to the genera *Combretum*, *Pteleopsis*, *Terminalia,* and *Quisqualis* for their antibacterial and antifungal effects. The background knowledge for this study was that a number of African species of Combretaceae are used to treat bacterial and fungal infections. Results from this screening indicated that *Pteleopsis myrtifolia* was not as active as the *Combretum* and *Terminalia* spp. for its antibacterial and antifungal effects. The lowest MIC values of 50 µg/mL was obtained by *C. imberbe* against *Staphylococcus epidermidis* and *C. elaegnoides* against *Shigella sonnei*. Moreover, *C. acutifolium*, *C. padoides*, and *C. nelsonii* displayed noteworthy activity against *B. cereus* (MIC 90–160 µg/mL). Compared to this, the lowest MIC for *Pteleopsis myrtifolia* was 750 µg/mL, against *B. cereus*. In addition, *C. imberbe*, *C. acutifolium,* and *C. elaegnoides* exhibited broad-spectrum antimicrobial activity with low average MIC values against both Gram-negative and Gram-positive bacteria. When the various genera were compared for their average MIC values, the genus *Combretum* exhibited the lowest value, followed by *Pteleopsis*. Moreover, according to a biochemometric analysis, the antimicrobial activity of those extracts displaying significant activity was related to their triterpene and flavonoid contents. Of the species screened in this investigation, *C. imberbe* is used commonly for diarrhea, while *C. zeyheri* and *C. apiculatum* are used for the treatment of bloody diarrhea. In summary, the results from these screenings especially justify the use of *Combretum* species for treatment of diarrhea.

In a study by Cock and Van Vuuren [29], methanol and water extracts of the leaves of two *Combretum* spp. and six *Terminalia* spp. used in South African traditional medicine for symptoms related to infections or infectious diseases were screened for their antibacterial and antifungal activities. Both the inhibition zone diameters (IZD) and MIC values were obtained with agar diffusion methods. All extracts showed broad-spectrum antibacterial activity, inhibiting the growth of 75–100% of the tested bacterial strains. Moreover, the Gram-positive and Gram-negative bacteria were approximately equally susceptible to the extracts. In general, the *Terminalia* species showed better effects than the *Combretum* species. The antibacterial effects of most of the extracts were mild, with MIC values ranging between 200 and 5000 µg/mL. For the *Combretum* species, the methanol extracts showed better activities than the water extracts against Gram-positive bacteria, whereas the opposite was true for the *Terminalia*. The authors attributed the good activity of the water extracts of *Terminalia* spp. to the high tannin content of these extracts. Moreover, when compared to the *Combretum* extracts, the *Terminalia* extracts were more effective against the Gram-negative bacteria. The best antibacterial effects were obtained with a water extract of *T. sericea,* which demonstrated an MIC value of 31 µg/mL against *B. cereus.* This result justifies the traditional medicinal use of macerations from the leaves of *T. sericea* to treat diarrhea. The best antifungal effects were obtained with methanol extracts of *C. molle*, which showed MIC values of 126, 172, and 259 µg/mL against *Aspergillus niger*, *Candida albicans,* and *Rhizopus stolonifera,* respectively. *T. sericea* methanol and water extracts showed the best antifungal effects of the tested *Terminalia* spp., with MIC values of 215 and 235 µg/mL against *R. stolonifer*.

In a comprehensive study, Eloff et al. [107] investigated acetone leaf extracts of twenty-seven species of the genera *Combretum*, *Terminalia*, *Pteleopsis* and *Quisqualis* for their antibacterial effects against *Staphylococcus aureus, Enterococcus faecialis, Pseudomonas aeruginosa* and *Escherichia coli*. All extracts inhibited the growth of both Gram-positive and Gram-negative bacteria. However, the effects differed largely between species and between freshly made and stored extracts (with six weeks of storage). In summary, the MIC values ranged from 0.1 to 6.0 mg/mL, with an average of 2.01 mg/mL. The mean MIC was 1.8 mg/mL against Gram-positive bacteria, while it was 2.22 mg/mL against the Gram-negative strains. The lowest MIC values were obtained with freshly made leaf extracts of *Quisqualis littoria* and *Combretum molle* (MIC < 0.1 and 0.2 mg/mL, respectively, against *P. aeruginosa*) and *Terminalia brachystemma* (MIC < 0.2 mg/mL against *S. aureus*), and with stored extracts of *Combretum padoides* and *Combretum nelsonii* against *Pseudomonas aeruginosa* (MIC < 0.1 mg/mL for both). *Terminalia sericea* showed rather low MICs of 0.4 mg/mL against *Enterococcus faecalis* and 1.2 mg/mL against *E. coli*. The antibacterial results for *C. molle*, *C. padoides*, and *C. nelsonii* could justify their use in African traditional medicine for the treatment of infections.

Fyhrquist et al. [36] combined an ethnomedical investigation on the medicinal use of Combretaceae plants in Mbeya, Tanzania, with a screening of the antimicrobial activity of extracts of *Combretum* and *Terminalia* species (hot water, methanol, acetone, and ethanol) against Gram-negative and Gram-positive bacteria and *C. albicans*. The screening methods used were the cylinder and the hole–plate agar diffusion methods. Almost all *Combretum* and *Terminalia* extracts were active against *Bacillus subtilis* and *S. aureus*, but only *T. kaiserana* demonstrated a growth-inhibitory effect against *E. coli* (bactericidal effect). Methanol extracts of the roots of *T. sambesiaca*, *T. kaiserana*, and *T. sericea* displayed the largest inhibition zone diameters (25–40 mm). When compared to the *Terminalia* spp., the *Combretum* spp. were slightly less active, with the largest inhibition zone diameter shown by a root methanol extract of *C. fragrans* against *Micrococcus luteus*. Moreover, both root and stem bark methanol extracts of *C. padoides* showed good growth-inhibitory effects against a number of bacteria, including *S. aureus* and *E. aerogenes*. Notably, many *Combretum* extracts were effective against the Gram-negative *E. aerogenes*. These results support the ethnomedical uses of the plants in the study for the treatment of infections and their symptoms.

In another study, Fyhrquist et al. [17] screened the antifungal effects of a large number of *Combretum* and *Terminalia* species against yeasts (*Candida* spp.) and *Cryptococcus neoformans*. The most active species by far were *T. sambesiaca* and *T. kaiserana*, with IZD values of 32 and 30.3 mm, respectively, of their methanol root extracts against *C. glabrata*. Methanol root extracts of *C. molle* and *C. padoides* were also particularly active against *C. glabrata,* with slightly smaller inhibition zone diameters than compared to *T. sambesiaca* and *T. kaiserana*.

Elegami et al. [74] studied the antibacterial and antifungal effects of extracts from *Combretum hartmannianum*, *Terminalia arjuna*, and *Combretum pentagonum*. These species were selected based on their ethnomedical uses in Sudan for the treatment of wounds, jaundice, and bronchitis. Aqueous extracts were prepared using the infusion method, which is one of the methods used for the preparation of traditional remedies from Combretaceae in African traditional medicine. Activities were determined using agar diffusion and dilution methods, and extracts providing inhibition zone diameters of 15 mm were considered active. The MIC values varied from 1.20 to 69.28 mg/mL. In terms of the MIC results, the *C. hartmannianum* methanol extracts of the leaves and water extracts of the fruits provided good growth inhibitory effects against *B. subtilis* (with MIC values of 1.43 and 1.91 mg/mL, respectively). The methanol extracts of the fruits and leaves of *T. arjuna* provided moderate growth inhibitory effects against *B. subtilis* and *S. aureus*, with MIC values of 2.89 and 2.43 mg/mL, respectively. Although *C. pentagonum* extracts provided rather high MIC values against the tested bacteria, methanol extracts of barks, leaves, and fruits resulted in large inhibition zone diameters (21–23 mm) against *B. subtilis*, *S. aureus*, and *E. coli*. The results of this investigation support the use of the screened plants for the treatment of wounds (*C. pentagonum*) and bronchitis (*T. arjuna*). Moreover, the antibacterial effects of the extracts were attributed to tannins and flavonoids.

In a comprehensive study, Masoko et al. [108] studied the antifungal effects against *Candida* species and *Cryptococcus neoformans* of 24 species of *Combretum*. Acetone–leaf extracts of *C. moggii* and *C. petrophilum* provided the lowest MIC value, 20 µg/mL.

## 5. Phytochemistry and Antimicrobial Compounds in *Combretum* and *Pteleopsis* spp.

Natural products have many modes of action relevant to antimicrobial potency. These modes of action include the inhibition of proteins, lipids, RNA, DNA, and cell-wall synthesis. Moreover, plant-derived compounds can disrupt the membrane integrity and coagulate the cell content. In addition, other modes of action include interference with microtubule function (for example, anti-tubulin effects), the inhibition of cell division, interference with ion uptake, the destabilization of the proton motive force (PMF), electron flow, active transport (drug efflux inhibition), reduction in protein secretion, dysfunction of RNA processing, and the inhibition of DNA methylation [6,143,144]. Plant-derived compounds also inhibit biofilm formation, bacterial motility and attachment, and the communication between microbial cells (anti-quorum sensing). Flavonoids (catechins and naringenin), ellagic acid, ellagic acid derivatives, and ellagitannins (cyclic-carbohydrate-containing ellagitannins, C-glycosidic ellagitannins with an open-chain glucose core, gallo-ellagitannins, and flavano-ellagitannins) are often considered to be important phytochemicals with antimicrobial activity within the Combretaceae family [12]. However, regarding the genera *Combretum* and *Pteleopsis*, only a few studies have been performed on ellagitannins and the ellagic acid derivatives and/or their antimicrobial activity, although more studies exist on the antimicrobial flavonoids in these genera. When considering the number of studies concerning antimicrobial compounds in *Combretum* and *Pteleopsis*, pentacyclic triterpenes have been evaluated in many studies, and low MIC values are reported for some of them [13,26]. Additionally, within the genus *Combretum,* a number of stilbenes have been characterized, of which some have demonstrated good antibacterial potential [145]. Table 5 summarizes some of the studies that have been made on the phytochemistry of African *Combretum* and *Pteleopsis* species with focus on antimicrobial compounds.

### 5.1. Phytochemistry and Antimicrobial Compounds of Combretum Species

Of the species of the genus *Combretum* (approximately 250 species), only thirty-one (31) species have been studied for their phytochemistry [15]. To date, at least 261 compounds, mainly terpenoids (of which the majority are triterpenes) and phenolic compounds (phenolic acids, diarylpropanes, tannins, flavonoids, stilbenoids, and phenanthrenes), have been isolated from *Combretum* species (Table 5). Simple triterpenoids and triterpenoid glycosides, as well as stilbenoids (such as combretastatins), are common in the genus *Combretum* [15]. Some of them, such as combretastatins B-5 and B-1 and their glycosides, as well as hydroxyimberbic acid, have shown potent antibacterial effects, with MIC values as low as 1.56–3.9 µg/mL [147]. In general, cycloartane, lupane, ursane, oleanane, and dammarane-type triterpenes are well-known in the *Combretum* species [15,68,168]. Other compounds include lignans, amino acids (non-protein), lipids, and steroids [15,28]. Moreover, though not widely found, some alkaloids have been identified in *Combretum* species [45]. An alkaloid extract of *C. zeyheri* showed efflux-pump inhibitory activity, although the active compounds were not characterized [135]. In addition, hydrolysable tannins, including ellagi- and gallotannins and condensed tannins (proanthocyanidins), have been characterized in African *Combretum* species [23].

#### 5.1.1. Triterpenes and Saponins

Triterpenes are involved in the antimicrobial defence system of many plants, and *Combretum* spp. accumulate them especially in the secretory glands (trichomes) of their leaves [169]. At least ten African species of *Combretum*, with a common occurrence in southern Africa and reputed uses for bacterial and fungal infections in traditional medicine, were studied for their antimicrobial triterpenes and triterpenoid glycosides (saponins) (Table 5). The species that have been studied most frequently are *C. imberbe* and *C. padoides* (syn. *C. minutiflorum* Exell). Moreover, *C. erythrophyllum*, *C. racemosum*, *C. vendae*, *C. zeyheri*, *C. collinum, C. molle*, and *C. laxum* were also studied in this respect [149,150,151]. Leaf material was used in most studies, but roots (*C. racemosum*) and stems (*C. laxum*) have also been used. As not only the leaves, but also the roots and stems of *Combretum* spp. are used for the preparation of herbal remedies for the treatment of infections, these parts of *Combretum* spp. should also be investigated for their triterpenes.

**Figure 4:**
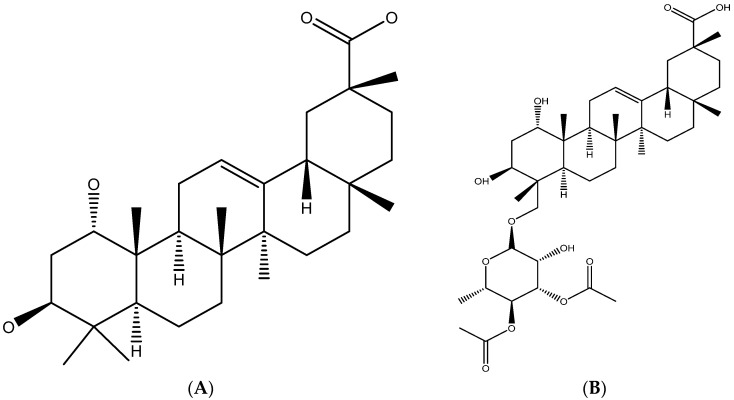
(**A**) The pentacyclic triterpene, imberbic acid, and (**B**) 23-hydroxyimberbic acid 23-O-α-L-3,4-diacetylrhamnopyranoside, a triterpene saponine. Both compounds were isolated from *C. imberbe* leaves [147]. Source: ChemDraw, SciFinder 2022.

Hydroxylated pentacyclic olean-12-ene triterpene saponins, as well as the triterpenoid aglycones, hydroxyimberbic acid, and imberbic acid, were isolated from the leaves of *C. imberbe* (Figure 4). The rhamnose-containing saponins inhibited the growth of a number of Gram-positive bacteria but were less active than imberbic acid [147]. Imberbic acid showed the lowest MIC values of 1.56 and 3.13 µg/mL against *Mycobacterium fortuitum* and *S. aureus*, respectively. However, all saponins and the imberbic acid were less active against *E. coli*, with MIC values above 100 µg/mL [147]. Mollic acid and its glucosides, including mollic acid–β-D-glucoside, -arabinoside and -xyloside, as well as imberbic acid, were characterized from the leaf trichomes of *C. molle* and *C. petrophilum* [148,169]. However, according to our literature review, mollic acid and its glucosides have not been tested for their antimicrobial effects; they are foremost known for their good molluscididal effects [169]. Dawe et al. [168] found two new cycloartane-type triterpenes, combretins A and B, from the leaves of *Combretum fragrans* (syn. *C. adenogonium*). Angeh et al. [26] isolated a new antibacterial oleanane-type triterpenoid glycoside from a dichloromethane extract of the leaves of *C. padoides*, namely 1α, 23β-dihydroxy-12-oleanen-29-oic-acid-23β-O-α-4-acetylrhamnopyranoside and 1,22-di-hydroxy-12-oleanen-30-oic acid. Both compounds showed activity against *S. aureus* and *E. coli* (with an MIC of 63 µg/mL for both compounds). The pentacyclic triterpene olean-12-ene-3-one, isolated from the leaves of *C. collinum*, was mildly antibacterial against *S. aureus* and *E. coli* (with an MIC of 568.9 µg/mL) [146].

#### 5.1.2. Flavonoids

A number of African *Combretum* species have been investigated for their flavonoids and/or the antimicrobial effects of isolated flavonoids (Table 5). Among the most studied African species regarding flavonoids are *C. micranthum*, *C. apiculatum*, and *C. erythrophyllum*. Various methoxylated and hydroxylated flavonoid derivatives, including quercetin derivatives, are common within the genus *Combretum* [170]. Flavonoids from African *Combretum* species have been found to inhibit the growth of bacteria and fungi and, in addition, some flavonoids were found to affect the quorum-sensing system of bacteria. For example, Vandeputte et al. [119] found that *C. albopunctatum*, a species indigenous to Madagascar, contains catechins that inhibited the transcription of quorum-sensing (QS) factor regulation genes in *P. aeruginosa*. Moreover, in a later study, Vandeputte et al. [171] found that naringenin, eriodictyol, and taxifolin, also isolated from *C. albopunctatum*, significantly reduced the QS-dependent production of pyocyanin and elastase in *P. aeruginosa,* without affecting its growth.

Kaempferol and the methoxylated quercetin derivatives rhamnocitrin, rhamnazin, and quercetin-5,3′-dimethylether, as well as genkwanin, apigenin, and hydroxy-4′,7-dimethoxyflavone, were characterized from an acetone leaf extract of *C. erythrophyllum* [60]. All the compounds were active against *Vibrio cholerae* and *Enterococcus faecalis* (with MICs of 25–50 µg/mL).

**Figure 5:**
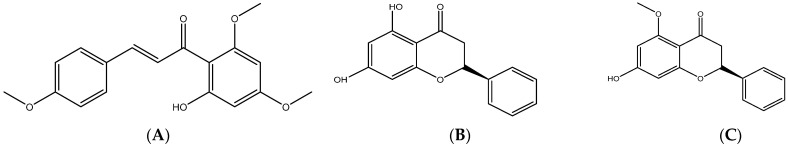
Structures of three antibacterial flavonoids known from African *Combretum*: (**A**) flavokawain A, (**B**) pinocembrin, and (**C**) alpinetin. ChemDraw, SciFinder 2022.

Katerere et al. [156] characterized two simple chalcones, cardamomin and 4′-hydroxy-2′,6′-dimethoxychalcone, from the leaves of *C. apiculatum*. The antibacterial effects of both compounds were moderate to weak (with MIC values 50–100 µg/mL), with *S. aureus* being more sensitive than *M. fortuitum* and *E. coli*. Additionally, both chalcones inhibited *C. albicans* with an MIC of 50 µg/mL. In addition, pinocembrin, alpinetin, and chrysin were characterized from the leaf extract of *C. apiculatum*. Pinocembrin showed strong growth inhibition against *C. albicans,* with an MIC of 6.25 µg/mL (compared to an MIC of 12.5 µg/mL for fluconazole) and good activity against *S. aureus* (with an MIC of 12.5 µg/mL). These MIC values were hitherto the lowest reported regarding flavonoids in African *Combretum* species. Katerere et al. [155] presented follow-up results on flavonoids in *C. apiculatum* leaves and isolated three antibacterial flavonoids: the phytoalexins flavokawain A, a chalcone originally found in *Piper methystichum* (Kava), and alpinetin and pinocembrin (Figure 5). *S. aureus* was the most sensitive bacterium and was inhibited at an MIC of 40 µg/mL by flavokawain A and alpinetin. However, pinocembrin demonstrated an MIC of 80 µg/mL against *S. aureus*; this contrasted with the MIC of 12.5 µg/mL that was established in the previous investigation by Katerere et al. [156]. *E. faecalis* was inhibited at an MIC of 40 µg/mL by alpinetin and pinocembrin. Pinocembrin and alpinetin inhibited *Pseudomonas aeruginosa* at an MIC of 40 µg/mL. Salih et al. [76] found that luteolin, which was present in a root ethyl acetate extract of *C. hartmannianum*, inhibited the growth of *M. smegmatis* at an MIC of 250 µg/mL. Moreover, in this same investigation, Salih et al. [76] found that also quercetin-3-O-galactoside-7-O-rhamnoside-(2→1)-O-β-D-arabinopyranoside was present in the antimycobacterial root extract of *C. hartmannianum*.

#### 5.1.3. Hydrolysable Tannins, Their Derivatives, and Condensed Tannins

Although hydrolyzable tannins (HT) such as gallotannins (GT), ellagitannins (ET) and their derivatives (ellagic acid and gallic acid derivatives) are common in *Combretum* spp., only seven species have hitherto been studied in depth regarding these compounds [15,172] (Table 5). This could be because most ETs that have been studied for their antimicrobial effects to date have shown moderate or mild in vitro growth inhibitory effects (with MIC values mostly around 25–1000 µg/mL, with some exceptions), and their bioavailability is poor when used in oral medications. Interestingly, however, ETs were found to potentiate the effects of antibiotics [173] and could have great potential especially in topical applications. In their review, Buzzini et al. [174] pointed out that, although the antimicrobial effects of hydrolysable tannins are well studied, most studies have not evaluated this activity.

**Figure 6:**
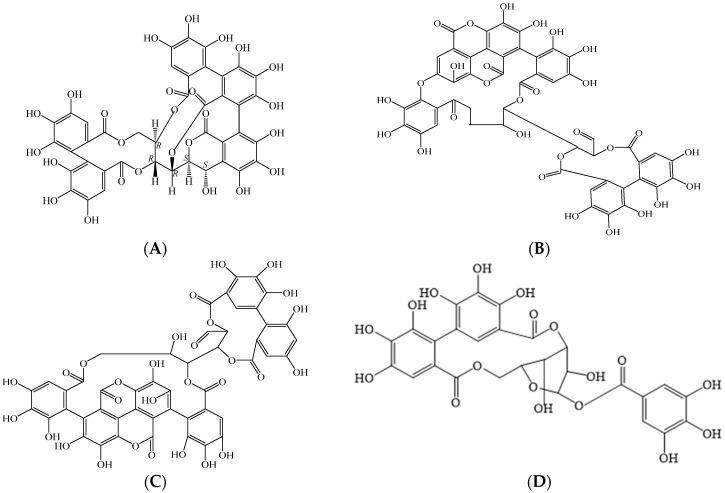
Ellagitannins in African *Combretum* spp. (**A**) castalagin, (**B**) terchebulin, (**C**) punicalagin, and (**D**) corilagin. ChemDraw, SciFinder 2022.

Polyphenols, and especially ellagitannins, are not well studied in *Combretum* spp. [15,172]. The molecular structures of some ellagitannins found in *Combretum* spp. are presented in Figure 6. Other genera and species, especially *Terminalia* spp. (Combretaceae), *Punica granatum* (Lythraceae), and *Eucalyptus* spp. (Myrtaceae)—all of which belong to the order Myrtales, which is rich in ETs—have been studied more thoroughly for their ellagitannins. Some of the ellagitannins found in these genera are also found in some *Combretum* spp., such as punicalagin and the ellagitannin monomer, 2,3-*S*-hexahydroxydiphenoyl-D-glucose, the characteristic component of many ETs [175,176,177]. Jossang et al. [176] were among the first to study ellagitannins in *Combretum* in 1994. They found that water decoctions of the leaves of *Combretum glutinosum* contained punicalin, punicalagin, 2,3-*S*-hexahydroxydiphenoyl-D-glucose, and combreglutinin. The ellagitannins were not tested for their antimicrobial effects in this study.

The extracts of many African *Combretum* spp. are also rich in proanthocyanidins (condensed tannins) and related polyphenols, such as epigallocatechin and catechin [23,178]. Tannins were found to be useful for the prevention of food spoilage and especially for topical applications, such as for the treatment of skin infections and wounds, as well as for mouthwashes and in toothpastes via their antimicrobial and antioxidative effects [122,179]. Moreover, tannins reduced the growth of pathogenic clostridia, but did not affect the probiotic lactobacilli and bifidobacteria in the gut [180]. In African countries, traditional medicinal preparations from *Combretum* spp., such as decoctions and macerations, are used both topically and orally for the treatment of infections. These preparations have been shown to be rich in hydrolyzable tannins, and especially ellagitannins, which could explain the antibacterial and antifungal potency of these species [23,65]. Thus, African *Combretum* species could be potential sources of tannin-enriched extracts and tannins for food safety and for the treatment of topical and oral infections, as well as for balancing the microbial flora in the gut. Some studies on tannins and their derivatives in African species of *Combretum* and their in vitro antimicrobial effects are discussed below, and more are listed in Table 5.

In two antibiotic assays, including a growth-inhibition assay in broth and a single-cell infection antibiotic assay using *Mycobacterium marinum* as test bacterium and *Acanthamoeba castellanii* as host, Diop et al. [65] found that α- and β-punicalagin, isolated from a decoction of *Combretum aculeatum*, possessed an IC_50_ value of 51.48 µM, compared to 6.99 µM for rifampicin. As α- and β-punicalagin are not bioavailable, Diop et al. [65] also studied the antibacterial effects of urolithins, the metabolites resulting from the metabolic degradation of α- and β-punicalagin, and found that urolithins A, B, and D provided weak growth-inhibitory effects against *Mycobacterium marinum*. However, due to the high content of tannins in the decoction of *C. aculeatum* for the treatment of TB, Diop et al. [65] suggested that the levels of urolithins might reach plasma concentrations that would be relevant for in vivo antimycobacterial effects. Thus, Diop et al. [65] concluded that their results could justify the use of *C. aculeatum* decoctions for the treatment of TB in Senegalese traditional medicine, and that the anti-TB effects of these decoctions are related to their ellagitannins and particularly to punicalagin and its urolithin metabolites. In contrast to Diop et al. [65], Asres et al. [111] showed that punicalagin, isolated from the stem bark of *Combretum molle*, possessed only weak growth-inhibitory effects against *M. tuberculosis typus humanus* ATCC 27294, although the growth inhibition was total at concentrations higher than 600 µg/mL. Thus, different species and strains of *Mycobacterium* may differ in their sensitivity to ellagitannins. Some authors have, however, chosen to study the content of ETs and their related derivatives (ellagic acid derivatives) in *Combretum* extracts with good antibacterial activity. For example, Fyhrquist et al. [23] showed that a methanol extract of the stem bark of *C. psidioides*, which demonstrated a good growth-inhibitory effect against *Mycobacterium smegmatis* (MIC 625 µg/mL), contained corilagin, sanguiin-H4, and punicalagin (Figure 6), along with thirteen unknown ellagitannins and methyl ellagic acid xyloside as the main component. In this same investigation, ellagitannin and ellagic acid (EA) derivative-rich extracts of *C. zeyheri* and *C. padoides* were also found to provide growth inhibitory effects against *M. smegmatis*. In addition, methyl ellagic acid, dimethyl-ellagic acid, and dimethyl-galloyl ellagic acid were characterized in *Combretum zeyheri*, and ellagic acid arabinoside and methyl ellagic acid xyloside were present in *C. padoides* [23]. Previously, it was demonstrated that ellagic acid derivatives have antimycobacterial potential. Ellagic acid derivatives isolated from the stem bark of *Terminalia superba*, such as 3,4′-di-O-methyl-ellagic acid-3′-O-β-D-xylopyranoside and 4′-O-galloyl-3,3′-di-O-methyl-ellagic acid -4-O-β-D-xylopyranoside, were strongly active against *Mycobacterium smegmatis* and *M. tuberculosis*, with MIC values between 4.88 and 9.76 µg/mL [181]. However, Fyhrquist et al. [23] found that ellagic acid itself was not very active against *M. smegmatis,* with an MIC of 500 µg/mL, and therefore the methylations and glycosylations of EA seem to be important for its antimycobacterial activity. Moreover, Fyhrquist et al. [23] tested the growth inhibitory effect of corilagin against *M. smegmatis* to assess the contribution of ETs to the antimycobacterial effects of the *Combretum* extracts. Corilagin gave only a weak antimycobacterial effect (an MIC of 1000 µg/mL). Therefore, it was suggested that the ellagitannins act in concert with each other as well as with other compounds present in the active extracts. However, Fyhrquist et al. [23] pointed out that other, unidentified ETs in the *Combretum* extracts should be quantified (proportion of the extract), isolated and tested to assess the final contribution of the ETs to the antimycobacterial effects of the extracts. In some other studies, corilagin revealed a good antibacterial effect against *S. aureus,* having an MIC value of 25 µg/mL, and inhibited the growth of methicillin-resistant *S. aureus* (MRSA) [182,183]. In addition, corilagin showed potentiating effects in combination with various β-lactam antibiotics, reducing their MIC values against MRSA [181]. Interestingly, it was found that corilagin reduced the synthesis of penicillin-binding protein 2a, thus decreasing the resistance of MRSA to β-lactam antibiotics [183,184]. Thus, corilagin could be effective against skin infections and wounds caused by *S. aureus*.

Wood and bark extracts of *Combretum hartmannianum* are used in the treatment of bacterial infections. Thus, Mohieldin et al. [122] studied the effects of a methanol extract of the stem bark of *C. hartmannianum* on *Porphyromonas gingvinalis*, a bacterium causing periodontal diseases. The extract resulted in both growth inhibition (with an MIC of 0.5 mg/mL) as well as in metalloproteinase 9 (MMP-9) inhibition. Moreover, the terchebulin that was found in the extract inhibited MMP-9 significantly, although its growth-inhibitory effects against *P. gingvinalis* were moderate (MIC 500 µg/mL). Another ET in the extract, flavogallonic acid dilactone, provided an MIC of 1000 µg/mL against *P. gingvinalis*. In summary, regarding punicalagin, terchebulin, and flavogallonic acid dilactone, it was found that all compounds inhibited the growth of *Helicobacter pylorii* and *Propionibacter acne,* with MIC values ranging from 125 to 250 µg/mL [78].

Salih et al. [76] found that methanol Soxhlet and ethyl acetate extracts of the root of *C. hartmannianum* provided growth-inhibitory effects against *Mycobacterium smegmatis*. These effects were partly attributed to ellagitannins and ellagic acid derivatives since these compounds were the main components in the extracts. Fifty-four polyphenols were characterized in the ethyl acetate extract. Among them were gallic acid, terflavin B and its two isomers, castalagin, corilagin, tellimagrandin I and its derivative, (*S*)-flavogallonic acid dilactone, punicalagin, epigallocatechin gallate (EGCG), and methyl-ellagic acid xylopyranoside (Figure 6). However, when tested alone, corilagin, gallic acid, and ellagic acid demonstrated high MIC values against *M. smegmatis* in this study (500–1000 µg/mL). Castalagin, which was present in the root of *C. hartmannianum* [76], was found to inhibit the growth of *E. coli* [185,186,187] as well as *Vibrio* strains and *Aeromonas sobria* [154]. Moreover, tellimagrandin I, also found in the *C. hartmannianum* root [76], inhibited *S. aureus*, *E. coli* and *Clostridiales perfringens* [186]. In addition, tellimagrandin I was reported to markedly reduce the MIC of β-lactam antibiotics in MRSA via its ability to decrease the synthesis of penicillin-binding protein 2a [183]. To date, however, no studies exist on the effects of castalagin, tellimagrandin I, or (*S*)-flavogallonic acid on mycobacteria.

Epigallocatechin gallate (EGCG) that Fyhrquist et al. [23] found in a butanol extract of the stem bark of *C. psidioides* showed antibacterial activity against both Gram-negative and Gram-positive bacteria, among them *Aeromonas* and *Vibrio* strains [154]. Moreover, EGCG affects the cell-wall integrity of *M. smegmatis* mc 2155 [153].

A large number of oligomeric procyanidines were found from a leaf extract of *C. mucronata*, but the antimicrobial effects of these condensed tannins were not investigated. However, the procyanidines were found to possess anthelminthic effects [152].

#### 5.1.4. Stilbenoids (Bibenzyles and Phenanthrenes)

**Figure 7:**
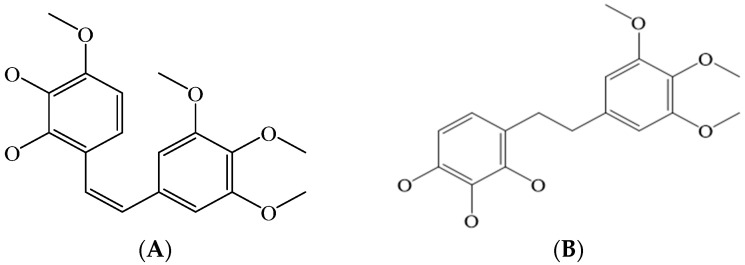

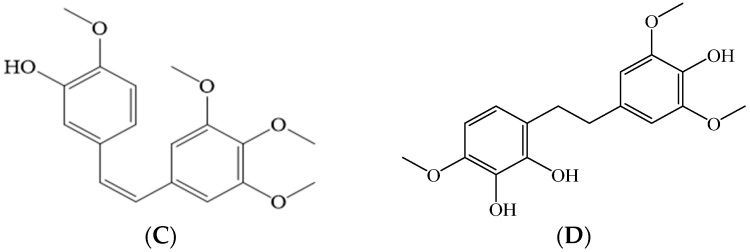
Stilbenes in *Combretum* spp. (**A**) combretastatin A-1, (**B**) combretastatin B-1, (**C**) combretastatin A-4, and (**D**) combretastatin B-5. ChemDraw, SciFinder 2022.

Bibenzyles and their derivatives are rare in higher plants. However, they are common within the genus *Combretum*; some of these compounds are listed in Table 5. In *Combretum* spp., cis-stilbenes (Combretastatins A), dihydrostilbenes (Combretastatins B), phenanthrenes (Combretastatins C, formed via phenolic oxidation of bibenzyls), and macrocyclic lactones (Combretastatins D) have been characterized [188]. Pure combretastatin was the first bibenzyl to be isolated from the fruits, stems, and barks of the South African tree *C. caffrum* [157]. Soon after, combretastatins A-2, A-3, and B-2 (CA-2, CA-3, and CB-2) were isolated from the stem wood of *C. caffrum* [158], and shortly after that, combretastatin A-1 and combretastatin B-1 (CA-1 and CB-1) were isolated [159]. Moreover, in 1988, the unusual combretastatin D-1, containing two additional rings (when compared to the A- and B-series combretastatins) and a lactone ring, was isolated from the stem wood of *C. caffrum* [160]. Additionally, the B-series combretastatins, combretastatin B-3 and B-4 (CB-3 and CB-4), were characterized from *C. caffrum* stem wood in 1988 [161]. In 1995, the most important bibenzyl compounds (with respect to their anti-cancer potential), namely, combretastatins A-4 (CA-4), A-5 (CA-5), and A-6 (CA-6), were characterized from the stem wood of *C. caffrum* [162]. Later, combretastatin A-4 (CA-4) was identified from *C. microphyllum* [189]. Additionally, combretastatin A-1, combretastatin B-1, and the corresponding 2-O-β-D-glucosides of the two combretastatins were identified from the root of *C. kraussii* [163] (Figure 7). Several stilbenoids were isolated from the wood of *C. erythrophyllum*: combretastatin A-1, (-)-combretastatin, combretastatin A-1-2-β-D-glucoside, and combretastatin B-1- 2-β-D-glucoside [190]. From the aerial parts of *C. molle,* 3,4′-dihydroxy-4,5-dimethoxybibenzyl and seven 1,9-dihydrophenantherens were characterized [166]. Stilbenes have also been characterized from *C. psidioides*, including fourteen 9,10-dihydrophenanthrenes and three bibenzyls (among them 4′-hydroxy-3,4,5-trimethoxybibenzyl) from the heartwood [191], as well as combretastatin B-2 from the stem bark [23].

Combretastatins are mainly known for their significant in vitro and in vivo anti-cancer effects via their inhibitory effects of tubulin polymerization and the disruption of tumor vasculature formation [192]. In this respect, combretastatin A-4 and A-1 specifically have been found to be some of the most potent, natural anti-tubulin compounds [192]. However, less is known regarding the antimicrobial potential of the combretastatins and phenanthrenes from *Combretum* species. Some of the combretastatins in *Combretum* spp. possess antibacterial effects. For example, combretastatin B-5, isolated from *C. woodii*, showed significant antibacterial activity against *Staphylococcus aureus* (with an MIC of 16 µg/mL) and lower activity against *Pseudomonas aeruginosa* and *Enterococcus faecalis* (with MIC values of 125 µg/mL) [13,145]. Combretastatins A-4 and A-5, isolated from *Combretum caffrum* stem wood, inhibited the growth of *Neisseria gonorrheae*, with MIC values ranging from 25 to 50 µg/mL [162]. Katerere et al. [156] found four phenanthrenes in a fruit extract of *C. hereroense* as well as one phenanthrene and two bibenzyls, including combretastatin, in a leaf extract of *C. collinum*. The phenantherenes were active against *Mycobacterium fortuitum* and *S. aureus* (with MIC values of 25 µg/mL), and apiculatol (a bibenzyl) was active against *C. albicans* and *S. aureus* (with MIC values of 25 µg/mL). Mushi et al. [164] isolated three substituted phenanthrenes from the root of *C. adenogonium*, of which all had significant growth inhibitory activity against *P. aeruginosa*. Malan & Swinny [165] isolated five substituted 9,10-dihydrophenanenthrenes and four phenanthrenes from the heartwood of *C. apiculatum*; three phenanthrenes provided complete growth inhibition against *Penicillium expansum* in a bioautography assay.

#### 5.1.5. Cyclobutanes

Katerere et al. [167] isolated two novel cyclobutane chalcone dimers from a dichloromethane extract of the aerial parts of *C. albopunctatum*. The compounds were not tested for their antimicrobial activities. However, heteroaryl chalcones containing a cyclobutane ring structure are known to possess antimicrobial effects [193].

#### 5.1.6. Alkaloids

**Figure 8:**
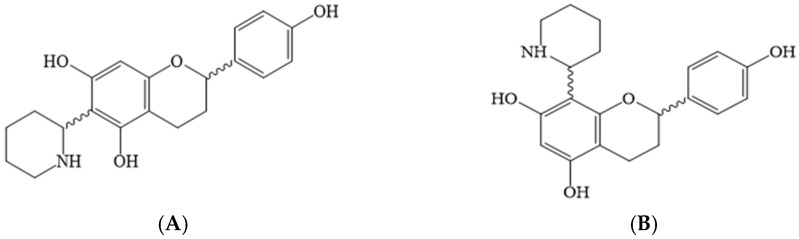
Piperidine-flavan alkaloids (kinkéloid- alkaloids) characterized from *Combretum micranthum* [45]: (**A**) kinkeloid A1 and (**B**) kinkeloid A2. ChemDraw, SciFinder 2022.

Only few alkaloids are known from *Combretum* spp. The pyrrolidine alkaloid, combretine, and the piperidine alkaloid betonicine have been isolated from the leaves of *C. micranthum*. In two different studies, a large number of piperidine-flavan alkaloids—the kinkeloids, with a completely new basic molecular structure consisting of a piperidine unit attached to the 6- or 8-carbon of the flavan backbone—were found in leaf extracts of *C. micranthum* (Figure 8) [27,45,194]. Kinkeloids of the A-, B-, C-, and D-series were characterized. Piperidine-flavan alkaloids are not known from other plants. The combination of a flavan unit with a piperidine alkaloid can make flavan-piperidine alkaloids particularly biologically active [194]. According to a qualitatitive screening of the alkaloids in a leaf extract of *C. dolichpetalum*, the extract contained quinolone, isoquinoline, tropane, purine, and indole alkaloids [195]. However, the molecular structures of these alkaloids were not further characterized.

### 5.2. Phytochemistry and Antimicrobial Compounds of Pteleopsis Species

GC-MS analyses of an aqueous extract of the stem bark of *P. suberosa* showed that the stem bark contained, inter alia, the following compounds: arjunglucoside (67.36%), taxifoline (7.42%), luteolin (4.88%), reserpine (3.72%), furoquinoline (3.51%), berberine (3.02%,), ursolate (2.23%), and cryptolepine (2.00%) [96]. De Leo et al. [95] isolated thirteen triterpenoids from chloroform, methanol and n-butanol extracts of the stem bark of *P. suberosa*, four of which were new triterpenoid glycosides. Moreover, the triterpenoids were tested for their anti-*Helicobacter* activities since *P. suberosa* stem bark decoctions are used in Malian traditional medicine for the treatment of ulcers. Additionally, a methanol extract of the stem bark had shown anti-*Helicobacter* effects in a previous investigation [142]. Arjunglucoside I was the only active triterpenoid among the thirteen tested. It significantly inhibited three metronidazole-resistant strains of *H. pylori*, with MIC values ranging from 1.9 to 7.8 µg/mL, the effects being comparable to clarithromycin and much more effective than metronidazole [95].

Pteleopsoside (syn. bellericagenin {B 3-O-[β-D-glucopyranosyl-(1→2)-α-D-glucopyranoside], a new saponin with two D-glucose units, and two sphingolipids, hylodendroside-II and I, were isolated from the stem bark of *Pteleopsis hylodendron*. In addition, 2α, 3β, 23-triacetoxy-19α-hydroxyolean-12-en-28-oic acid, friedelin, lupeol, β-carotene, sitosterol, and stigmasterol were characterized [196]. Sphingolipids were found to protect the liver from toxic compounds and to have antitumor, antimicrobial, and immunostimulatory effects [197,198].

Ngounou et al. [90] isolated two new saponins (2α, 3α, 19α, 23-tetrahydroxyolean-12-en-28-O-β-D-galactoside and a triterpenoid (2α, 3β, 21β, 23-tetrahydroxyolean-12-en-28-oic acid) from the stem bark of *P. hylodendron*. The structures of these compounds were elucidated by spectroscopic studies.

Fractions containing anthraquinones, alkaloids, and anthocyanins were isolated from a methanol extract of the stem bark of *P. hylodendron.* Two of these fractions inhibited the growth of *E. coli*, *Proteus mirabilis*, *Salmonella paratyphi* B, *Enterococcus faecalis*, and *S. aureus* with an MIC of 0.97 µg/mL, compared to MIC values of 781–12,500 µg/mL for the crude methanol extract [139]. In contrast, all the fractions containing only alkaloids were devoid of activity.

## 6. Potentiating Effects

In African traditional medicine, herbal decoctions and other preparations commonly consist of a combination of two or more medicinal plants [36,199,200]. Moreover, in traditional, small-scale farming in African countries, combinations of plants are also used as extracts for crop-plant protection [201,202]. The various phytochemicals in the extracts can enhance the antimicrobial effects of each other and lead to synergistic and/or additive effects [203]. It has been demonstrated that plant extracts and plant-derived compounds can act synergistically and/or additively with conventional antimicrobial drugs [7]. Using plant-derived compounds/extracts as antibiotic adjuvants could be a means of reducing the required doses of antibiotics, thus reducing their adverse health effects and at the same time restoring the potency of antibiotics that have lost their effects against resistant strains of bacteria and fungi. Plant extracts and their compounds could be used as antibiotic adjuvants, especially against multi-resistant bacteria and fungi. The interest in the potential of plant extracts and plant-derived compounds as antibiotic adjuvants is a growing research field and was recently proposed by many researchers [204]. However, not all extracts and compound combinations result in synergistic effects, and it is therefore important to calculate the fractional inhibitory concentration index (FICI) that defines the nature of the combination effect (synergistic, additive, indifferent, or antagonistic) [19]. For the genus *Pteleopsis*, our literature search resulted in no findings on combination studies with antibiotics or with extracts of other medicinal plants.

### 6.1. Combination Effects of Combretum Species with Antibiotics and Other Plant Extracts

To date, a small number of studies have been performed on the interactions of extracts of the African *Combretum* species with conventional antimicrobials (antibiotic-resistance modifying effects) and/or with other plant species. Most of these interaction studies were performed using microdilution methods, and some studies included a checkerboard method. Agar diffusion was used as a screening method in some studies. In most of the screenings, the plant extracts and antibiotic combinations, as well as the extracts’ combinations, displayed synergistic effects.

Table 6 summarizes the results reported in the literature on the combination effects of extracts of *Combretum* species with of antibiotics and with extracts of other medicinal plants on bacterial and fungal growth. For example, *C. edwardsii* and *C. kraussii* have been studied in this respect. They were found to produce strong synergistic effects with many antibiotics, including the third-generation cephalosporine and cefotaxime. Drug-resistant *S. aureus* was especially sensitive, whereas drug-resistant *E. coli* and *K. pneumoniae* showed more resistance [30]. Interestingly, none of the combinations were found to be antagonistic. Additionally, a water extract of *C. kraussii* in combination with penicillin showed especially good growth inhibition against *S. aureus* with a FICI value of 0.04, indicating a strong synergistic effect [30].

In a comprehensive study by Fankam et al. [205], extracts of *Combretum molle*, *Allanblackia gabonensis*, and *Gladiolus quartinianus* were screened for their interaction effects with conventional antibiotics on the growth of Gram-negative bacteria, including drug-resistant phenotypes of *E. coli*, *Enterobacter aerogenes*, *Klebsiella pneumoniae*, *Pseudomonas aeruginosa*, and *Providencia stuartii*. Antibiotic-modulating effects, ranging from 67–100% for methanol leaf extracts of *C. molle* in combinations with chloramphenicol, kanamycin, streptomycine, and tetracycline, were observed against multi-resistant bacteria, and a 64-fold reduction of the MIC of streptomycin alone was observed in combination with streptomycin against a multi-drug-resistant strain of *E. coli*.

The synergistic antimicrobial effects of leaf extracts of *Combretum hereroense* in combination with leaf extracts of the *Citrus lemon* and *Apodytes dimidiata* (Metteniusaceae) species were investigated against *Mycobacterium smegmatis* using a microdilution method [31]. The MICs of the plant combinations ranged from 0.04 mg/mL to 1.25 mg/mL, when compared to the MICs of 0.1–3 mg/mL for the extracts when tested alone. The combinations that provided the lowest MIC of 40 µg/mL, i.e., those of an acetone extract of *Apodytes diminata* and a hexane extract of *Combretum hereroense* as well as a combination of dicloromethane and methanol extracts of the aforementioned species, indicated that the herbal combination was better than the single-plant-species extracts at inhibiting the growth of *M. smegmatis*.

In a recent study by Seepe et al. [19], a large number of plant species, including *Combretum erythrophyllum* and *C. molle*, were screened for their individual and combination effects against plant-pathogenic *Fusarium* species. When screened individually, extracts of *Combretum erythrophyllum*, *Harpephyllum caffrum*, and *Quercus acutissima* were the most active, with MIC values smaller than 0.1 mg/mL. *C. erythrophyllum* showed synergistic or additive effects against all tested *Fusarium* strains in combinations with *Solanum mauritianum*, *Harpephyllum caffrum*, *Quercus acutissima*, *Nicotiana glauca*, *Withania somnifera*, and *Schotia brachypetala.* Combined acetone extracts of *Harpephyllum caffrum* and *C. erythrophyllum* showed strong, synergistic antifungal activity against *F. graminearum*, *F. proliferatum*, and *F. verticillioides* (MIC values of 0.02 mg/mL, 0.002 mg/mL and 0.001 mg/mL, respectively). In addition, a combination of the ethyl acetate extracts of the leaves of *C. molle* and *Nicotiana glauca* showed strong synergistic effects against *Fusarium proliferatum*. Antagonistic effects were detected for some plant extract combinations, such as the ethyl acetate and acetone extracts of *C. molle* and *C. erythrophyllum* against *F. proliferatum*. The plants were selected based on their previously reported activity against animal and/or human fungal pathogens. In summary, this study indicates that combinations of plant extracts are good alternatives to conventional, synthetic fungicides and supports the established use of extract combinations in African traditional medicine.

### 6.2. Nature and Significance of Interactions

Antimicrobial resistance and the adverse effects of antibiotics can be reduced in two different ways, among other things: by combining antimicrobial plants with each other or by combining antimicrobial plant extracts, fractions, or compounds with antimicrobial drugs [31]. Given that the development of new drug therapies for the treatment of infectious diseases is time-consuming and expensive, it is worth the effort to try different types of combination therapies [206,207]. Numerous studies have shown that, in combination with antibiotics, plant extracts and plant-derived compounds increase the activity of antibiotics and, when allowing for the use of smaller doses of the antibiotics, reduce the side effects caused by antibiotics. Indeed, these positive interactions are considered a potential strategy in the fight against bacterial antibiotic resistance, as phytochemicals often act through different mechanisms than conventional antibiotics and could therefore be useful in the treatment of infections caused by resistant bacteria. According to current knowledge, plant-derived compounds modulate and inhibit bacterial resistance mechanisms (e.g., the overexpression of efflux pumps, drug inactivating and target-modifying enzymes, and the transformation of permeation barriers) and thus exhibit synergistic effects with conventional antibiotics [6,208].

### 6.3. Putting Synergies into Practice

In African traditional medicine, the interactions of the numerous compounds in ointments and other herbal preparations made from medicinal plants are utilized when applied topically for the treatment of wound infections and inflammations on the skin. Not only are essential oils often used in different combinations, but plant extracts are also used in combination with each other; for example, in the treatment of skin diseases, in order to improve the effect [209]. The antifungal potential of the crude extracts of selected *Combretum* and *Terminalia* species and a mixture of asiatic acid and arjunolic acid isolated from *Combretum nelsonii* (syn. *C. kraussii*) was confirmed in a study which examined the in vivo antifungal effects of plant extracts and compound combinations on cutaneous wound healing in immunosuppressed rats [83]. *Combretum imberbe*, *Combretum nelsonii*, and *Combretum albopunctatum*, used in the study by Masoko et al. [83], contain large concentrations of tannins and other polyphenolic compounds that have a broad spectrum of antimicrobial activity against skin-related pathogens, supporting the use of these medicinal plants for dermatological diseases. Moreover, tannin-rich extracts of *Combretum* and *Pteleopsis* could contain tannins with beneficial effects on the bacterial flora in the gut. Most ellagitannins metabolize to urolithins in the gut, and these metabolites have been considered to have a beneficial effect on health-promoting intestinal bacteria while reducing the growth of harmful Clostridia [210]. Ahmad et al. [211] suggested that the methanol leaf extract of *Combretum hypopilinum* could affect the adrenergic systems in the antidiarrheal activity. The stem bark n-butanol fraction of *Pteleopsis suberosa* had anti-ulcer effects when it was tested with ethanol-induced gastric ulcers in rats and carrageenan-induced paw edema in mice [25].

## 7. Conclusions

Although many *Combretum* and *Pteleopsis* species are utilized in African traditional medicine, the research and knowledge of the mechanisms underlying the antimicrobial effects of these plants and their compounds, as well as their synergistic effects with each other and with antibiotics, is still ongoing and incomplete. A new generation of standardized and effective antimicrobial preparations cannot be developed without comprehensive information on the antibacterial and antifungal potential of the extracts and the compounds they contain. In addition, the in vivo testing of activity, toxicity, and bioavailability determines the true role of extracts and compounds from *Combretum* and *Pteleopsis* spp. in the treatment of human infectious diseases.

Although not always used as a selection criterion for antimicrobial screenings, ethnopharmacological knowledge has played a significant role in finding extacts and compounds with good antimicrobial potential from *Combretum* and *Pteleopsis* species. Significantly, the potent antimicrobial activities of preparations mimicking traditional remedies, such as macerations and decoctions, have in many cases confirmed the claimed uses in traditional medicine for the treatment of infections. Altogether, it has been confirmed that a number of extracts and compounds from the African species of *Combretum* and *Pteleopsis* have promising antimicrobial potential. The most significant antimicrobial activities among the pure compounds were shown by imberbic acid (with an MIC of 1.56 µg/mL against *Mycobacterium fortuitum*), arjunglucoside I (with an MIC of 1.9 µg/mL against drug-resistant *Helicobacter pylorii*), and pinocembrin (with an MIC of 6.25 µg/mL against *C. albicans*).

Screenings on the interactions of extracts of *Combretum* species with conventional antibiotics or with other plant species are sparse. Furthermore, there are no reported studies on the combination effects of *Pteleopsis* species with other plant species or with conventional antibiotics. However, some studies indicate that extracts of certain species of *Combretum* show strong synergistic effects with conventional antibiotics. Of special notice are the promising potentiating effects of the extracts of *Combretum molle* on streptomycin against antibiotic-drug-resistant *E. coli*. Compounds or standardized extracts of *C. molle* might have future uses for drug repurposing against drug-resistant bacteria. So far, no pure compounds from African *Combretum* spp. have been evaluated for their combination effects with antibiotics. However, a mixture of asiatic acid and arjulonic acid, isolated from *C. nelsonii* (syn. *C. kraussii*), was very active against *Candida* species and *Cryptococcus neoformans*, with MIC values between 0.2 and 1.6 µg/mL.

In combination with other plants, as they are used in traditional medicine or for crop-plant protection, *Combretum* species show significant synergistic effects, against both human-pathogenic bacteria and some plant-pathogenic fungi. This justifies the customary use of *Combretum* species in combinations with other plants for the treatment of infections and for crop plant protection in African traditional medicine and agriculture. Moreover, using extract combinations to combat resistant bacteria and fungi could overcome the problem of the development of antibiotic resistance due to the multiple components in the plant extracts.

It is interesting to note that there are no reports on the scientific evaluation of the antimycobacterial effects of *Combretum erythrophyllum* and *C. micranthum*, though both species are used in traditional medicine for the treatment of tuberculosis. Thus, in-depth studies on the extracts of these plant species and their bioactive compounds should be conducted to ascertain their reported use in the treatment of coughs and tuberculosis in Guinean and African traditional medicine.

## Figures and Tables

**Table 4:** Examples of antimicrobial screenings including *Combretum*, *Pteleopsis*, *Quisqualis,* and *Terminalia* species (Combretaceae) to compare the antimicrobial effects within and between genera.

Plants	Parts of Plants and Extracts	Bacterial and Fungal Strains	MIC Value Range and Most Active Extracts	Reference
Fifty-one species were screened: thirty-nine *Combretum* spp., two *Pteleopsis* spp. and nine *Terminalia* spp.	Leaves; methanol	*Staphylococcus aureus*, *Bacillus cereus*, *Staphylococcus epidermidis*, *Enterococcus faecalis*, *Escherichia coli*, *Shigella sonnei*, *Salmonella typhimurium*, *Pseudomonas aeruginosa*, and *Klebsiella pneumoniae*	MIC range: from 0.05 to >3.00 mg/mL; MIC 0.05 mg/mL for *C. elaeagnoides* and *C. imberbe* against *Salmonella enterica* and *Shigella sonnei,* respectively. *Combretum* species more active than *Terminalia* spp. and *Pteleopsis myrtifolia.*	[109]
*C. glutinosum*, *C. hispidum*, *C. molle*, *C. nigricans*, *P. suberosa*, *T. avicennioides*, and *T. mollis*	Leaves, shoot, and stem bark Ethyl alcohol–water (50:50, *v*/*v*)	*Candida albicans*, *Epidermophyton floccosum*, *Microsporum gypseum*, *Trichophyton mentagrophytes*, and *Trichophyton rubrum*	MIC range: from 0.25 to >4 mg/mL; 0.25 mg/mL for many species against *Epidermophyton* and *Trichophyton*. *P. suberosa* most active.	[46]
*C. collinum*, *C. erythrophloeum*, *C. erythrophyllum*, *C. hereroense*, *C. microphyllum*, *C. molle*, *T. prunioides*, and *T. sericea*	Leaves; water, and methanol	Twelve Gram-negative rods, two Gram-positive rods, two Gram-positive cocci, and three fungi	MIC range: 0.031–6 mg/mL; 0.031 mg/mL for a water extract of *T. sericea* against *B. cereus.* *Terminalia* species were the most active.	[29]
Twenty-two *Combretum* species, *P. myrtifolia*; three *Terminalia* species (*T. branchystemma*, *T. prunioides*, and *T. sericea*); and *Quisqualis littoria*	Leaves; acetone	*Staphylococcus aureus*, *Escherichia coli*, *Pseudomonas aeruginosa*, and *Enterococcus faecalis*	MIC range: 0.1–6 mg/mL. Freshly made leaf extracts: MIC 0.2 mg/mL for *T. brachystemma* and *C. molle* against *S. aureus* and *P. aeruginosa*, respectively, and MIC 0.1 mg/mL for *Q. littoria* against *P. aeruginosa.* Stored leaf extracts: MIC 0.1 mg/mL for *C. padoides* and *C. nelsonii* against *P. aeruginosa.*	[107]
*C. apiculatum*, *C. collinum*, *C. constrictum*, *C. fragrans*, *C. hereroense*, *C. molle*, *C. obovatum*, *C. padoides*, *C. psidioides*, *C. zeyheri*, *P. myrtifolia*, *T. kaiserana*, *T. sambesiaca*, *T. sericea*, *T. spinosa*, and *T. stenostachya*	Leaves, roots, fruits, stem bark; acetone, ethanol, methanol, and water	*Candida albicans*, *Candida krusei*, *Candida tropicalis*, *Candida glabrata*, *Candida parapsilosis*, and *Cryptococcus neoformans*	IZD from 0 mm to 32.2 mm. Best results: *T. sambesiaca*, root, 32 mm against *C. glabrata*; *T. kaiserana,* root, 30.4 mm against *C. glabrata.* *C. padoides* and *C. molle* methanol root extracts gave good growth inhibition, but were slightly less active compared to the *Terminalia* species. *P. myrtifolia* was not as active as the *Combretum* and *Terminalia* spp.	[17]
*C. fragrans*, *C. hereroense*, *C. molle*, *C. padoides*, *C. psidioides*, *C. zeyheri*, *T. kaiserana*, *T. sambesiaca*, *T. sericea*, and *T. stenostachya*	Leaves, roots, stem bark, fruits; methanol, acetone, ethanol, and water	*Staphylococcus aureus*, *Escherichia coli*, *Enterobacter aerogenes*, *Staphylococcus epidermidis*, *Bacillus subtilis*, *Micrococcus luteus*, *Sarcina* sp., and *Candida albicans*	IZD from 0 mm to 40 mm. Best results: Root extracts of *T. sambesiaca*, *T. kaiserana*, *T. sericea*, and *C. fragrans.*	[36]
*C. imberbe*, *C. nelsonii*, *C. albopunctatum*, and *T. sericea*	Leaves; Acetone	*Candida albicans*, *Cryptococcus neoformans*, *Microsporum canis*, *Sporothrix schenckii*, and *Aspergillus fumigatus*	MIC range from 0.02 to 0.64 mg/mL Best result: *C. nelsonii* and *T. sericea* leaf extracts with an average MIC of 0.16 mg/mL.	[83]
Twenty-four *Combretum* species	Leaves; acetone, hexane, DCM, and methanol	*Candida albicans*, *Cryptococcus neoformans*, *Aspergillus fumigatus*, *Sporothrix schenckii* and *Microsporum canis*	MIC range from 0.02 to >2.5 mg/mL Best result: methanol extracts of *C. moggii* and *C. petrophilum*, lowest MIC 0.02 mg/mL.	[108]

Abbreviations: MIC—minimum inhibition concentration; IZD—diameter of inhibition zone—DCM, dichloromethane.

**Table 5:** Compounds in African species of *Combretum* and *Pteleopsis* and their antimicrobial potential.

Species	Compounds	Antimicrobial Activity	References
	**Triterpenes/triterpenoids and saponins**		
*C. collinum* (leaf)	Olean-12-ene-3-one	Antibacterial activity against *S. aureus* and *E. coli* with an MIC of 568.9 µg/mL.	[146]
*C. imberbe* (leaf)	Imberbic acid, 1α,3β-hydroxyimberbic acid-23-O-α-L-4-acetylrhamnopyranoside, and 1α,3β,23-trihydroxy-olean-12-en-29-oic acid-23-O-α-[3,4-diacetyl]- rhamnopyranoside	An MIC of 1.56 µg/mL against *Mycobacterium fortuitum;* an MIC of 3.13 µg/mL *S. aureus;* an MIC of 12.5 µg/mL *S. aureus;* an MIC of 12.5 µg/mL *M. fortuitum;* and an MIC of 6.25 µg/mL *S. aureus.*	[147]
*C. imberbe* (leaf)	1α,3β-dihydroxy-12-oleanen-29-oic 1-hydroxy-12-olean-30-oic acid, 3,30-dihydroxyl-12-oleanen-22-one, 1,3,24-trihydroxyl-12-olean-29-oic acid, and 1α,23-dihydroxy-12-oleanen-29-oic acid-3β-O-2,4-di-acetyl-L-rhamnopyranoside	MIC between 16 and >250 µg/mL against *S. aureus*, *E. faecalis*, *P. aeruginosa,* and *E. coli.*	[26]
*C. molle* (leaf)	Mollic acid–3-O-β-D–glucoside and imberbic acid	Mollic acid–3-O-β-D–glucoside was not tested for antimicrobial effects by Pegel & Rogers [148], but Katerere et al. [147] showed that imberbic acid inhibits *M. fortuitum* and *S. aureus.*	[147,148]
*C. nelsonii syn. C. kraussii* (leaf)	Asiatic acid and arjunolic acid (in antifungal TLC fractions from an acetone extract; bioautography)	MIC of a mixture of asiatic acid and arjunolic acid was between 0.2 and 1.6 µg/mL against *C. albicans*, *Cryptococcus neoformans*, *Microsporum canis*, *Sporothrix schenckii*, and *Aspergillus fumigatus.*	[82]
*C. padoides* (leaf)	Triterpenoid desmosides, oleanane-type triterpenoid glycosides, and (25(27)-dehydroporiferasterol	Several of these compounds showed significant antibacterial activity against *S. aureus* and *E. coli.*	[149]
*C. racemosum* (root)	Several triterpenoids: (1) 28-O-β-D-glucopyranosyl-2α,3β,21β,23-tetra- hydroxy-olean-18-en-28-oate (3) Arjungenin (5) Terminolic acid (11) 3-acetyl ursolic acid (14) Betulinic acid (15) Quadranoside II	Compound 1: MIC values of 128 and 256 µg/mL against *E. coli* and *E. faecalis.* Compounds 3, 5, and 11: MIC values between 64 and 256 µg/mL against *S. aureus*, *E. coli* and *E. faecalis.*	[150]
*C. zeyheri* (leaf)	Ursolic acid, oleanolic acid, maslinic acid, 2α,3β-dihydroxy-urs-12-en-28-oic acid, 6β-hydroxymaslinic acid, and terminolic acid	All compounds showed anti-*Candida* activity of which terminolic acid was most active; MIC values between 62.50 and 125 µg/mL against strains of *C. albicans.*	[151]
*P. suberosa* (stem bark)	Ten oleanane-based saponins and three aglycones; arjunglucoside I, arjunglucoside II, sericoside, sericic acid, arjunetin, trachelosperogenin, bellericoside, and arjungenin	Arjunglucoside I was active against *H. pylori* (ATCC 43504) and its metronidazole-resistant strains (Ci 1 cag A, Ci 2 vac A, and Ci 3) with MIC values between 1.9 and 7.8 µg/mL.	[95]
	**Tannins (hydrolysable and condensed and related derivatives)**		
*C. hartmannianum* (bark)	Terchebulin and flavogallonic acid	MIC values of 500 and 1000 μg/mL against *Porphyromonas gingivalis.*	[122]
*C. hartmannianum* (root)	Terflavin B, terflavin B-isomer I, terflavin B-isomer II, corilagin, (*S*)-flavogallonic acid dilactone, tellimagrandin I, α-punicalagin, terchebulin (or β-punicalagin), and tellimagrandin I derivative	MIC of 313 and 625 µg/mL, respectively, for a methanol Soxhlet extract and an ethyl acetate extract of the roots against *Mycobacterium smegmatis.*	[76]
*C. molle* (stem bark)	Punicalagin	Punicalagin totally inhibited the growth of *M. tuberculosis typus humanus* ATCC 27294 at concentrations higher than 600 µg/mL.	[111]
*C. mucronatum* (leaf)	A large number of condensed tannins; epicatechin (1) and oligomeric proanthocyanidins (OPC) 2–10	A dose-dependent anthelmintic activity ranging from 1 to 1000 μM.	[152]
*C. psidioides* (stem bark)	Epigallocatechin gallate (EGCG)	Present in a butanol extract of the stem bark that showed antimycobacterial activity against *Mycobacterium smegmatis*. In another study [153], EGCG showed inhibition of the cell-wall integrity of *M. smegmatis*. Antibacterial against stains of *Aeromonas* and *Vibrio* [154].	[23,153,154]
*C. zeyheri, C. padoides* and *C. psidioides* (stem bark and root)	Corilagin, punicalagin, sanguiin H-4, and methyl ellagic acid xyloside as the main components in *C. psidioides* stem bark; ellagic acid arabinoside and ellagic acid xyloside in *C. padoides* stem bark; punicalagin, methyl-ellagic acid-xyloside, di-methyl-ellagic acid xyloside, and 3,3′-Di-O-methyl-4-O-(n′′-O-galloyl-β-D-xylopyranosyl)- ellagic acid in *C. zeyheri*	MIC values against *Mycobacterium smegmatis*: Corilagin: 1000 µg/mL Ellagic acid: 500 µg/ml	[23]
	**Flavonoids**		
*C. albiflorum*	Catechins	Inhibit transcription of quorum-sensing related genes.	[119]
*C. apiculatum* (leaf)	Flawokawain A (4′-hydroxy-2′,6′- dimethoxychalcone), pinocembrin (5,7- dihydroxyflavanone), and alpinetin (5-methoxy-7-hydroxyflavanone)	The isolated flavonoids were moderately active against *S. aureus* and *E. faecalis* at MICs of 40 µg/mL.	[155]
*C. apiculatum* (leaf)	(1) 5,7-dihydroxyflavanone (pinocembrin) (2) 2′,4′-dihydroxy-6′-methoxychalcone (cardamomin) (3) 5-hydroxy-7-methoxyflavanone (alpinetin) (4) 5,7-dihydroxyflavone (chrysin)	(1) MIC at 12.5 µg/mL against *S. aureus* and MIC at 6.25 µg/mL against *C. albicans;* (2) MIC from 100 µg/mL or higher against *E. coli*, *Mycobacterium fortuitum*, *Proteus vulgaris,* and *S. aureus* and MIC at 50 µg/mL against *C. albicans;* (3) MIC at 25 µg/mL against *C. albicans* and 100 µg/mL against *Proteus vulgaris* and *S. aureus;* (4) MIC at 50 µg/mL against *C. albicans* and 100 µg/mL against all bacteria tested.	[156]
*C. erythrophyllum* (leaf)	Apigenin, genkwanin, 5-hydroxy-7,4′-dimethoxyflavone, rhamnocitrin, kaempferol, quercetin-5,3′-dimethylether, and rhamnazin	MIC values in the range of 25–50 μg/mL against *Vibrio cholerae* and *Enterococcus faecalis*; rhamnocitrin and quercetin-5,3′-dimethylether inhibited *Micrococcus luteus* and *Shigella sonnei* at an MIC of 25 μg/mL.	[60]
*C. hartmannianum* (root)	Luteolin and quercetin 3-O-galactoside-7-O-rhamnoside –(2→1)-O-D-arabinopyranoside	Luteolin showed a growth-inhibitory effect against *M. smegmatis* (with an MIC of 250 µg/mL)	[76]
	**Stilbenes**		
*C. apiculatum* (leaves)	5-hydroxy-3,4′-dimethoxybibenzyl; 4′- hydroxy-3,4,5-trimethoxybibenzyl; 4′, 5-dihydroxy-3,4-dimethoxybibenzyl, and 4,4′-dihydroxy-3,5-dimethoxybibenzyl	5-hydroxy-3,4′-dimethoxy- bibenzyl was active against *C. albicans*, *Proteus vulgaris* and *S. aureus* with an MIC of 25–50 µg/mL. 4′-hydroxy-3,4,5-trimethoxy- bibenzyl was active against *C. albicans* with an MIC of 50 µg/mL.	[156]
*C. caffrum* (branches, leaves and fruits used in a combination extract)	Combretastatin	Combretastatin caused astrocyte reversal and inhibited the murine P388 lymphocytic leukemia cell line. No antimicrobial tests.	[157]
*C. caffrum* (stem wood)	The cis-stilbenes, combretastatin A-2 (CA-2), combretastatin A-3 (CA-3), and the trans-stilbene, combretatastatin B-2	Combretastatins A-2 and A-3 inhibited markedly the polymerization of tubulin in P388 lymphocytic leucemial cells. No antimicrobial tests.	[158]
*C. caffrum* (stem wood)	The cis-stilbene, combretastatin A-1 (CA-1), was isolated for the first time, and combretastatin B-1 was obtained by selective hydrogenation of CA-1.	Combretastatins A-1 and B-1 inhibited microtubule assembly in vitro and where potent inhibitors of the binding of colchisin to tubulin. No antimicrobial tests.	[159]
*C. caffrum* (stem wood)	The unusual macrocyclic lactone, combretastatin D-1, was isolated from a species of *Combretum* for the first time.	Combretastatin D-1 showed PS (P388 lymphocytic leukemia), cell-line inhibitory activity at ED_50_ 3.3 µg/mL. No antimicrobial tests.	[160]
*C. caffrum*	Combretastatin B-3 and B-4	PS leukemia ED_50_ values of 0.4 and 1.7 µg/mL, respectively. No antimicrobial tests.	[161]
*C. caffrum* (stem wood)	Combretastatin A-4, A-5 and A-6	Growth-inhibitory effect against *Neisseria gonorrheae:* CA-4 and CA-5: MIC between 25 and 50 µg/mL, CA-6: MIC between 50 and 100 µg/mL.	[162]
*C. kraussii* (root)	Combretastatin A-1 and B-1 and their corresponding 2-O-β-D-glucosides	Growth-inhibitory effect against mouse lymphocytic leukemia cells. No antimicrobial tests.	[163]
*C. psidioides* (stem bark)	Combretastatin B-2 and its dihydrostilbene derivatives were present in a methanol extract of *C. psidioides* stem bark.	MIC not tested for CB-2, but MIC for the MeOH extract of the stem bark of *C. psidioides* against *M. smegmatis* was 625 µg/mL.	[23]
*C. woodii* (leaf)	2′,3′,4-trihydroxyl-3,5,4′-trimethoxybibenzyl (combretastatin B-5)	MIC between 16 and >250 µg/mL against *S. aureus*, *E. coli*, *P. aeruginosa,* and *E. faecalis.*	[145]
	**Phenantrenes**		
*C. adenogonium* (root)	Substituted phenanthrenes	Compounds were active against *P. aeruginosa* with an MIC of 160 µg/mL.	[164]
*C. apiculatum* (heartwood)	Five substituted 9,10-dihydrophenanenthrenes and four phenanthrenes	Three phenanthenes totally inhibited the growth of *Penicillium expansum* at 20 µg/mL in a bioautographic analysis.	[165]
*C. collinum* (leaf)	9,10-dihydro-3,6,7-trimethoxy-2,5- phenanthrenediol	Active against *M. fortuitum* and *S. aureus* with an MIC of 25 μg/mL.	[156]
*C. hereroense* (fruit)	(1) 5,7-dimethoxy-1,2,3-phenanthrenetriol (2) 5,7-dimethoxy-2,3-phenanthrenediol (3) 9,10-dihydro-3,5-dimethoxy-2,7-phenanthrene- diol (4) 3,5,7-trimethoxy-2,6-phenanthrenediol	Compounds 1, 2, and 3 showed some activity against *M. fortuitum* and *S. aureus* with an MIC of 25 μg/mL.	[156]
*C. molle* (heartwood)	Fourteen 1,9- dihydrophenanthrenes and three phenolic bibenzyls	No biological activity tests.	[166]
	**Cyclobutanes**		
*C. albopunctatum* (aerial parts)	Two novel cyclobutane chalcone dimers	No tests.	[167]
	**Alkaloids**		
*C. dolichopetalum* (root)	Echinulin and arestrictin B (indole containing diketopiperazine alkaloids)	No tests.	[34]
*C. micranthum*	Piperidine-flavan alkaloids from n-butanol extracts of the leaves: Kinkeloid A1, A2, B1, B2, C1, C2, D1, and D2	No tests.	[27,45]

Abbreviations: MIC—minimum inhibitory concentration; CA-1—combretastatin A-1; CA-2—combretastatin A-2; CA-3—combretastatin A-3; CA-4—combretastatin A-4; CA-5—combretastatin A-5; CA-6—combretastatin A-6; CB-1—combretastatin B-1; CB-2—combretastatin B-2; CB-3—combretastatin B-3; CB-4—combretastatin B-4; CB-5—combretastatin B-5; CD-1—combretastatin D-1; and ED_50_—effective dose resulting in 50% growth inhibition.

**Table 6:** Antibiotic and extract potentiating effects in two-component combinations. Reported interactions of *Combretum* species with antimicrobials and with extracts of other plants.

Species, Extracts and Antibiotics Combinations	Screening Method and Antibiotic Potentiating Effect; FICI, Reduction of MIC	Reference
***Combretum edwardsii*** Hexane, dichloromethane, and ethyl acetate fractions of *Combretum edwardsii* leaves + cefotaxime, ampicillin, chloramphenicol, penicillin and amoxicillin	**Checkerboard method**: **Drug-resistant *E. coli***: Synergistic effect of a hexane fraction and cefotaxime. FICI value of 0.07. **Multidrug resistant *Klebsiella pneumoniae***: Synergistic effects of hexane, dichloromethane, and ethyl acetate fractions with cefotaxime. FICI values between 0.03 and 0.12. A combination of the ethyl acetate extract and cefotaxime provided the strongest synergistic effects with an FICI value of 0.03. **Penicillin-resistant *Staphylococcus aureus***: Synergistic effects of hexane, dichloromethane, and ethyl acetate fractions in combination with ampicillin, chlorampenicol, penicillin, and amoxicillin. FICI values between 0.05 and 0.37. The most effective combination was that of a hexane extract and amoxicillin, showing an FICI value of 0.05.	[30]
***Combretum kraussii*** Hexane, dichloromethane, ethyl acetate, and water fractions of *Combretum kraussii* leaves + cefotaxime, ampicillin, chloramphenicol, penicillin and amoxicillin	**Checkerboard method:** **Drug-resistant *E. coli***: Synergistic effects of dichloromethane and ethyl acetate extracts in combination with cefotaxime. FICI values of 0.07 and 0.064, respectively. The most effective combination was that of an ethyl acetate extract and cefotaxime. **Multidrug resistant *Klebsiella pneumoniae***: Synergistic effects of hexane, dichloromethane, ethyl acetate, and water fractions in combination with cefotaxime. FICI values of 0.062–0.38. The most effective combination was that of a hexane extract and cefotaxime. **Penicillin resistant *Staphylococcus aureus***: Synergistic effects of hexane, dichloromethane, ethyl acetate, and water extracts in combination with ampicillin, amoxicillin, chloramhenicol, and penicillin. FICI values between 0.04–0.38. The most effective combination was a water extract and penicillin.	[30]
***Combretum hereroense*, *Citrus lemon*, and *Apodytes dimidiata*** Hexane, dichloromethane, acetone, and methanol extracts of the leaves in two-species extract combinations	**Serial microdilution method:** The MICs of the crude extracts against *Mycobacterium smegmatis* ranged between 0.1 mg/mL (dichloromethane extract of *Apodytes dimidiata*) and 3 mg/mL (hexane extract of *Citrus lemon*). The MICs of the *C. hereroense* crude extracts ranged between 0.6 and 1.6 mg/mL, with the acetone and dichloromethane extracts being the most growth inhibitory. The best combinations; *Combretum hereroense* with *Apodytes dimidiata*, hexane and acetone, and dichloromethane and methanol; resulted in MIC values of 0.04 mg/mL and showed synergistic effects.	[31]
***Combretum erythrophyllum, Combretum molle, Harpephyllum caffrum, Quercus acutissima*, and *Solanum mauritianum*** Water, ethyl acetate, and acetone extracts of the leaves in two-species extract combinations	**Microplate dilution assay and FIC-index calculation:** MIC values of 0.04– > 2.5 mg/mL of the crude extracts of *C. erythrophyllum* against the tested *Fusarium* spp., with the strongest effects shown by the acetone and ethyl acetate extracts (MIC values of 0.04–0.08 mg/mL). Strong synergistic effects of the acetone extract of *C. erythrophyllum* in combination with acetone extracts of *Harpephyllum caffrum, Quercus acutissima,* and *Solanum mauritianum* against *Fusarium proliferatum* and *F. verticillioides* (MIC valuess of 0.002–0.001 mg/mL). MIC 0.04– > 2.5 mg/mL of the crude extracts of *C. molle* against the *Fusarium* spp. All tested extracts, including the water extracts, showed strong inhibition against *F. proliferatium* and *F. solani* (an MIC of 0.04 mg/mL). The ethyl acetate extract of *C. molle* demonstrated a strong synergistic effect in combination with an ethyl acetate extract of *Nicotiana glauca* against *Fusarium proliferatum* (an MIC of 0.001 mg/mL). Strong synergistic effects of the water extract of *C. molle* with a water extract of *Withania somnifera* against *Fusarium proliferatum* (an MIC of 0.002 mg/mL). Synergistic effects of acetone extracts of *C. molle* with acetone extracts of *Quercus acutissima* (an MIC of 0.001 mg/mL) against *F. proliferatum*.	[19]
***Combretum molle*** Methanol extract of the leaves + kanamycin and streptomycin	**Antibiotic modulation assay using a microdilution method:** At subinhibitory concentrations (MIC/2 and MIC/4) the leaf–methanol extract of *C. molle* resulted in a two- to sixty-four-fold increase of the antibacterial effects of kanamycin and streptomycin against Gram-negative bacteria (e.g., *E. coli, Enterobacter aerogenes, Pseudomonas aeruginosa, Klebsiella pneumoniae,* and *Providencia stuartii*), including multidrug-resistant clinical strains. No FIC index values were calculated.	[205]

Abbreviations: FICI—fractional inhibitory concentration index that indicates the quality of the interaction (synergistic, additive, intermediate, or antagonistic); and MIC—minimum inhibitory concentration.

## Data Availability

Not applicable.
